# Indigenous food environment and dietary patterns of Munda community of Jharkhand, India

**DOI:** 10.1186/s40795-025-01159-2

**Published:** 2025-10-21

**Authors:** Suparna Ghosh-Jerath, Ridhima Kapoor, Naina Gandhi, Swati C. Nair, Aman Rastogi, Arpita Ghosh

**Affiliations:** The George Institute for Global Health India, 308, Third Floor, Elegance Tower, Plot No. 8, Jasola District Centre, New Delhi, 110025 India

**Keywords:** Dietary patterns, Food environment, Indigenous communities

## Abstract

**Background:**

Indigenous communities in India are experiencing nutrition transition and facing its health and nutritional consequences. Exploring dietary patterns of one such community, the Munda tribes, can provide crucial information on how diverse food environment is translating into their dietary patterns.

**Methods:**

A cross-sectional study was conducted in 201 households of the Munda community of Jharkhand, India. Household surveys elicited information on socio-demographic and food access profile while a pre-tested food frequency questionnaire was used to assess dietary intake at HH level in monsoon (*n* = 160 HH) and winter (*n* = 100 HH). Factor analysis was used to derive dietary patterns and associations were explored between dietary patterns, food environment, and socio-demographic factors.

**Results:**

Three dietary patterns, each for monsoon and winter seasons, were identified in Munda community. These included “natured-procured dietary pattern” (majority of foods procured from nature like farms, forests, and water sources), “market-dominant dietary pattern” (majority of foods from the market) and “mixed-source dietary pattern” comprising indigenous and non-indigenous foods accessed from diverse sources, including natural food environments and markets Upon exploring the associations of dietary pattern scores with sociodemographic and food environment factors, it was seen that households with higher food access diversity scores had 3.93 times the odds of consuming more “mixed-source dietary pattern” (*p*-value < 0.05) during monsoon season. For the winter season, families living in nuclear family setup had significantly higher odds of consuming more market-dominant and nature-procured dietary patterns (*p*-value < 0.05), as compared to joint/extended families.

**Conclusion:**

The study provides valuable insights regarding the reliance of indigenous communities on natural food sources and their food procurement behaviour from markets. The socio-demographic matrix in terms of resource pooling was associated with market-dominant dietary patterns, whereby households accessing diverse food sources demonstrated higher score of mixed-source dietary patterns. The study can inform current policies and programs to address nutrition transition and mainstream indigenous wisdom and knowledge to improve diets of nutritionally vulnerable indigenous communities.

**Supplementary Information:**

The online version contains supplementary material available at 10.1186/s40795-025-01159-2.

## Introduction

Assessment of long-term and habitual dietary intake can objectively inform the role of diet in health and disease [1, 2]. The proportions, compositions, and combinations of different foods and nutrients in the diet, along with their frequency of consumption and interactions between different components of the diet, are referred to as a dietary pattern [2–4]. This dietary pattern,, has a significant influence on health and disease conditions. Unhealthy dietary patterns, like those predominantly rich in refined carbohydrates, fats, and salt, with reduced intake of micronutrients, have surpassed other lifestyle risk factors (such as tobacco use and alcohol consumption) to become the major cause of global mortality [5–8].

Nutrition epidemiology as a discipline strives to identify methods to objectively capture dietary patterns to inform role of diet in health and disease [9, 10]. The food frequency questionnaire (FFQ) is one such tool used extensively in epidemiological studies that enlists a variety of food items along with frequency of consumption while accounting for day-to-day variation in eating habits over a sufficient period of time [5–9]. To infer about the dietary patterns followed by communities, the data obtained from FFQ are translated into index-based parameters, majority of which use scores derived from food group recommendations and guidelines. However, these subjective scores do not accurately describe the overall dietary patterns of a community [11]. Recently, data-driven methods have been recognized as a novel approach to dietary pattern assessment and analysis [11, 12]. Principal Component Analysis (PCA) is a common data-driven statistical method used for this purpose [13]. Factor analysis using PCA technique can help analyse large datasets of food intake including data from FFQs by deducting dietary variables and grouping correlated food groups into uncorrelated factors i.e. dietary patterns [14]. This increases the interpretability of data by reducing its dimensionality and maximizing variance thereby minimizing the information loss from data [13].

In India, studies from diverse geographical regions have identified distinct dietary patterns amongst populations across different age groups (like children, adults, and adolescents). Some of these dietary patterns include ‘plant-based diets’ (roots and tubers and other vegetables), ‘traditional diets’ (pulses and legumes and condiments and spices), ‘micronutrient-rich diets’ (green leafy vegetables and fruits), ‘aquatic food-based diets’ (fish and sea-foods), ‘lacto-vegetarian’, ‘prudent pattern’ (non-starchy vegetables, fruits and milk and yoghurt), ‘snack and fruit pattern’ and ‘western dietary patterns’ like ‘starchy sugars’, ‘sugary drinks’ and ‘red-meat and high-fat dairy pattern’ [15–19]. Associations have also been drawn between these dietary patterns and nutritional outcomes; for instance, a study in China found that with westernization of diets, the ‘meat-diet structure’ among urban school students (between 11–17 years) was associated with an increased prevalence of obesity [20]. Another study reported that a diet predominantly consisting of rice, meat, sea foods and coconut oil-based dishes was positively associated with overweight and high blood glucose levels among women in rural India [21]. On the other hand, a lacto-vegetarian dietary pattern with limited intake of fruits and vegetables was linked with increased anaemia prevalence among rural Indian men [21].

Food environment, which is the physical, socio-economic and cultural context in which people interact with food systems and grow, acquire and consume food from a range of food access points, plays a critical role in influencing food choices of communities thereby affecting their dietary patterns [22, 23]. Communities living amidst natural food environment have historically accessed foods from farmlands, forests, and water bodies thereby reflecting diverse dietary patterns [24, 25]. Over the years, the food environments have become diverse with accessibility to foods from both natural food sources and markets which could have both healthy and unhealthy attributes [26]. Foods available in local food outlets, supermarkets, and multi-national food joints, apart from some minimally processed foods, also have a considerable share of packaged and processed foods rich in salt, fats, and sugar [26–28]. The improved accessibility, affordability and strong promotional strategies for packaged market foods is leading to nutrition transition with diets comprising predominantly packaged foods, ultra-processed foods, and starchy cereals [26, 29]. This nutrition transition is evident even in the nutritionally vulnerable and economically disadvantaged communities in low and middle-income countries (LMICs), which are reporting higher intakes of nutrient-poor, energy-dense ultra-processed foods while still struggling with the challenges of food insecurity. This has been attributed to the double burden of malnutrition in these communities including the Indigenous communities in India [30]. These communities are demonstrating co-existence of chronic energy deficiency and poor intake of essential micronutrients (iron and vitamin A) as well as increasing prevalence of diet-related non-communicable diseases [31, 32].

Distinct dietary patterns have been identified in indigenous communities that are undergoing nutrition transition [33]. India is home to several indigenous communities that constitute about 8.9% of the total Indian population and are identified as Scheduled Tribes or STs [34, 35]. The Indian tribal communities are deeply rooted in their historical cultures and primarily use “traditional and indigenous foods” that belong to their natural habitats and are accessed predominantly by farming or wild harvesting [36]. These indigenous foods are a good source of many nutrients, especially micronutrients like vitamin A, vitamin C, iron, zinc, folate, and calcium [37–39]. Tribal people constitute one-fourth of the total population of Jharkhand (26.2%), a central eastern state of India, known for its rich biodiversity and presence of natural resources [34, 40, 41]. Munda community is the third most populous tribal community in the state. The community has extensive traditional ecological knowledge (TEK) about its indigenous food systems consisting of diverse indigenous foods like wild plants, local fishes, mushrooms, tubers, and grains [42, 43]. Despite this, the community still has high levels of malnutrition [44, 45]. Apart from accessing food from their natural food environment, Munda community has been increasingly accessing food from the local markets and the supplementary feeding programs of the Government [43]. However, recent drivers related to agricultural intensification and mechanization, industrialization, climate change, outmigration and market proliferation, have collectively resulted in loss of TEK and traditional practices among Indian tribal communities [43, 46, 47]. Consequently, tribes like Munda, are experiencing nutrition transition, characterized by reduced consumption of traditional foods and increased reliance on market-procured ultra-processed foods, high in salt, sugar and fat [43, 45].

To our knowledge, there is a paucity of data on dietary patterns of nutritionally vulnerable indigenous communities who are undergoing a nutrition transition and are facing its health and nutritional consequences. Hence, exploring the dietary patterns of the Munda community can provide crucial information on how accessibility to diverse food environments is translating into their dietary patterns [42]. The present study aims to assess the dietary patterns of Munda indigenous community and explore their association with the community’s food environment and other socio-economic and demographic factors.

## Methodology

This work is part of a larger exploratory study on indigenous food (IF) systems of tribal communities of Jharkhand and understanding their impact on the nutritional status of tribal women and children. A detailed study protocol of the project is reported elsewhere [48].

### Study design and locale

We report a cross-sectional study that was conducted in two blocks (sub-district level administrative unit) of Khunti district in Jharkhand, India. To capture seasonality in dietary intake pattern, the study had a longitudinal component and data was collected in two seasons (monsoon season in August 2019 and winter season in January 2020). Hence, the dietary assessment tool was developed for these two seasons and listed food items available perennially or in specific seasons. This helped in capturing specific dietary patterns for monsoon and winter seasons.

### Sampling framework

We used a two-stage cluster sampling design where-in the two blocks in Khunti district, namely, Murhu and Torpa, were purposively selected based on ease of accessibility, geographical representation, and high population density of Munda tribal community. Using probability proportional to size (PPS) sampling, eleven villages in selected blocks (six from Murhu and five from Torpa) were identified from the tribal village list obtained from India’s Census conducted in 2011. In the second stage of cluster sampling, a sampling frame of all eligible household (HH) in the Munda community based on the larger study objectives were selected. These included HHs that had at least one non-pregnant adult woman in reproductive age group (18–49 years) and one child (6–54 months). Detailed sampling framework is provided elsewhere [49].

### Sample size calculation

The sample size calculation was based on the overarching objective of the larger study, which aimed to assess the contribution of IFs to the nutrient intake of tribal women and children of Jharkhand [49]. Thus, the sample size was calculated utilizing the iron intake data from a previous study among Santhal women in Jharkhand [50]. Based on a mean difference in dietary intake of 3.4 mg/day (SD = 7 mg/day) (consumer and non-consumer of IFs), 80% power, 5% significance level and design effect of 2, 134 women were required to be sampled in each group. This resulted in a total sample size of 268 women. A complete HH listing (*N* = 1005) was carried out in all the study villages to identify HHs with non-pregnant women (age group of 18–49 years). In a total of 201 eligible HHs where HH survey was completed, FFQs were administered in 160 HHs and 100 HHs in monsoon and winter season respectively. Forty-nine HHs were covered for FFQ across both the seasons.

### Study procedures

 Core principles of community-based participatory approach were integrated throughout the study including, development of study tools and data collection, to uphold ethical integrity of research. While developing the study tools, the Munda tribal youth were encouraged to participate in free-listing enquiries, which collectively informed the development of FFQ. The enumerators for data collection included Indigenous community members (from Munda tribe), who provided crucial support in interpreting the local language, and contextualizing the survey content to the study community. This active engagement of Munda community in the research was crucial for respecting their rights and interests in research, and maintaining ethical integrity [51].

#### Household survey

The HH survey elicited information on socio-demographic profile and access to different food sources. Information on food accessed from the natural food environment including cultivated food access points (like farmlands and kitchen gardens), wild food access points (like forests, wastelands and water bodies) and the built food environment (like local markets, ration shops, fair price shops etc.) were captured [22]. A pretested computer-assisted personal interviewing (CAPI) HH survey questionnaire was developed for this study (Supplementary file 1) on CS Pro software (Version 7.2) that was administered by trained field investigators on all eligible HHs using the Samsung tab (Model SM-T385).

### Assessing household-level diets

The FFQ was administered on a sub-sample of the population covered under the HH survey to elicit information on dietary intake at the HH level. Trained nutritionists administered these FFQs which were developed as paper forms. The seasonal FFQ for monsoon and winter season included specific food items accessed in the two seasons. To develop the FFQs, focus group discussions (FGDs) were conducted with the study community until data saturation. A free listing exercise was conducted to list all locally consumed food items and a directory was developed. Qualitative analysis of the FGD transcripts formed the basis for selecting food items for the FFQ [43]. Based on the seasonal availability of food items, a 283-item FFQ for monsoon season and a 232-item FFQ for winter season was developed for the present study (Supplementary file 2). The foods listed in the FFQs were further classified as indigenous and non-indigenous foods. Questions were systematically asked regarding the frequency of consumption of all the listed foods in the FFQ over the past 1 month. Since this was not a semi-quantitative FFQ, portion sizes were not asked. The predefined frequency categories ranged from least consumption i.e. “never during the past month” to most frequent consumption i.e. “2 or more times per day” and included a total of nine categories.

### Data management

In order to effectively manage the high-quality data, quality checks were ensured during data collection and at the data entry and analysis level. Standardized protocols for collecting socio-demographic and dietary data were followed by the field investigators, who were provided modular training. The data collected through the CS Pro app on the tablet had many built-in checks to maintain data quality. This data collected on the tablet was exported in an Excel sheet. The dietary data collected on paper-based FFQ were manually entered into the Excel sheet, and any outliers or incorrect/duplicate entries were removed. All the data was stored on password-protected laptops to maintain data security and prevent confidentiality breaches.

### Data analysis

#### Food accessed diversity index (FADI)

Based on the community’s knowledge regarding their food access from natural food environments (including both cultivated and wild food access points), an index was calculated that involves division of the number of foods obtained from natural food environments by a particularHHh (n) by the corresponding maximum possible number of foods grown, gathered, accessed, and raised in a particular village (N). The FADI was thus expressed as (n/N)^2^. The FADI scores were calculated directly by applying formula into the excel sheet.

#### Household wealth index

The socio-economic status of the population is a critical indicator that is likely to have an association with dietary intake. HH assets were utilized to assess the overall socio-economic status of the HH and included variables like type of house, number of rooms, source of electricity and drinking water, presence of separate kitchen and toilet, possession of another house, kitchen ware and monthly expense on food items. PCA technique was applied on these variables to derive an index called the HH Wealth Index.

#### Dietary patterns

The cleaned data entered in the excel sheet was re-categorised-for monsoon season FFQ data, 283 food items were clubbed into 24 broad categories of food groups, and for winter season data, 232 food items were clubbed into 23 broad categories. For PCA, these preliminary categories of food groups were then sub-categorized into 64 and 60 categories of foods for monsoon and winter season respectively (Supplementary file 3). This was based on community’s preference for food groups (commonly consumed/less commonly consumed), variety of the food item (indigenous/non-indigenous^1^*), food access point (cultivated food environment/wild food environment/market/Targeted Public Distribution System) and fatty acid composition of fats and oils. These food categories along with the frequency of intake data were then finalized for PCA.

### Statistical analysis

**Fig. 1:**
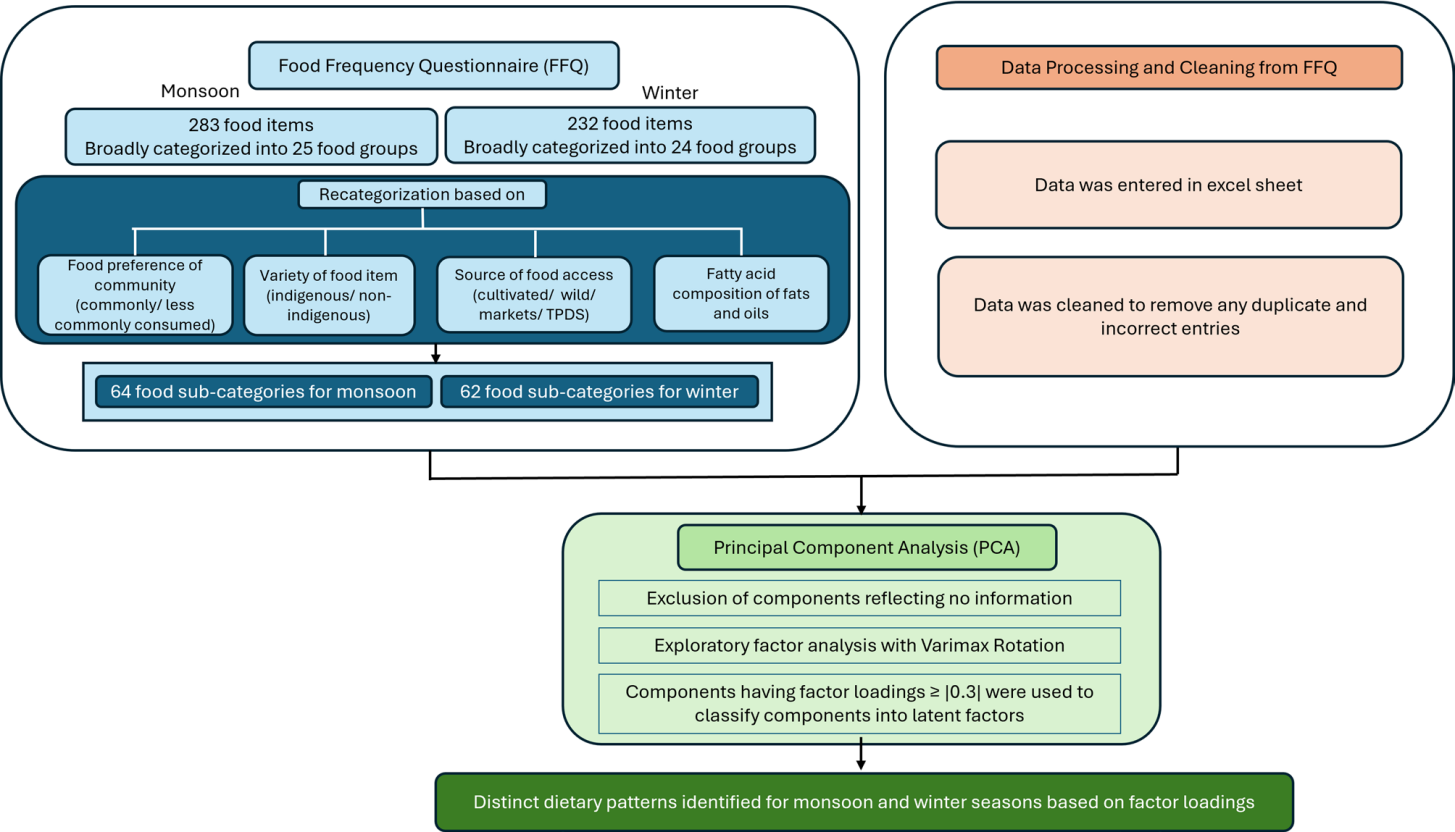
Methodology for FFQ data management and principal component analysis to identify dietary patterns

Univariate analysis was used to describe the sociodemographic characteristics of the HHs. All the categorical variables representing the socio-economic and food access profile were summarized using frequencies and percentages. All the continuous variables including wealth index and FADI were summarized using mean and standard deviation. Factor analysis was used to derive dietary patterns based on 64 and 60 food sub-categories, made from 283 item FFQ for monsoon season and 232 item FFQ for winter season respectively. Varimax rotation was used to rotate the factors to increase interpretability, and total extracted factor variance was not used to limit their number. Categories having factor loading < 0.3 were excluded while interpreting identified factors. The derived factors (dietary pattern) were named based on factor loadings (Fig. 1). Logistic regression was applied on the categorized dietary patterns (based on tertiles) to test the association of selected sociodemographic variables (like wealth index, type of family, number of family members, education and occupation of head of HHs, and ration card) and food access variables, for both seasons on the odds ratio scale. R version 4.3.2 software was used to conduct univariate and factor analysis.

### Determination of dietary patterns

The independent factors identified after PCA, were qualitatively analysed considering the loadings contributed by re-sorted food sub-categories. The food sub-categories which contributed ≥ |0.3| loading to the factor were considered to have a significant influence on the factor. These factors were considered to be independent dietary patterns. Food categories which had positive factor loading (≥ 0.3) signified that a particular dietary pattern predominantly consisted of a greater number of foods from those categories. On the other hand, food categories which had negative factor loading (≥ -0.3) signified that food items from these categories had lesser influence on that dietary pattern. Based on this analysis, the latent factors were given the name of a distinct dietary pattern for both the seasons (monsoon and winter). A total of three factors each, for both monsoon and winter season were identified.

## Results

This study was conducted on 201 HHs of eleven villages in the two blocks of Murhu and Torpa in Khunti district of Jharkhand. In this section, we report seasonal HH dietary patterns of the Munda community, while providing information on their sociodemographic and food access profile. Finally, associations between socio-demographic and food environment parameters with the seasonal dietary patterns is reported.

### Sociodemographic profile

As shown in Table 1, in majority (78%) of the HHs, the head of the HH was a male member. More than one-third (41%) of the study participants lived in nuclear families. About half (48%) of the heads of the HH had no formal education and majority (80%) of them had settled agriculture as their main source of income.

**Table 1:** Sociodemographic and food access profile of the households

Characteristic	*n* (%)
**SOCIODEMOGRAPHIC PROFILE** (***N* = 201**)	
**Gender of the Head of the Household**	
Male	156 (77.6)
Female	45 (22.3)
Family Type*	
Joint/Extended	119 (59.2)
Nuclear	82 (40.8)
**Education Level of the Head of the Household**	
No formal education	97 (48.3)
Primary schooling and below	60 (29.8)
Secondary schooling and above	44 (21.9)
**Occupation of the Head of the Household**	
Settled agriculture	160 (79.6)
Other	41 (20.4)
**FOOD ACCESS PROFILE**	
**Involved in Agricultural Practices** (*n*** = 200**)	
On own land	185 (92.5)
On leased land	7 (3.5)
Kurwa (Shifting Cultivation)	8 (4.0)
**Market access for food** (*n*** = 199**)	199 (100)
**Distance of the HH from the nearest market** (*n*** = 200**)	
<1 km	31 (15.5)
>5 km	67 (33.5)
1–3 km	65 (32.5)
3–5 km	37 (18.5)
**Collection of food items from forest** (*n*** = 200**)	144 (72.0)
**Distance of the HH from forest** (*n*** = 144**)	
<1 km	42 (29.2)
>3 km	72 (50.0)
1–3 km	30 (20.8)
**Pond access for collecting food items** (*n*** = 199**)	146 (73.4)
**Possession of livestock** (*n*** = 199**)	178 (89.5)
**Family size (Mean ± SD)** (*n*** = 201**)	5.9 ± 2.2
**Food Access Diversity Index (Mean ± SD) (** **n** **-201)**	0.3 ± 0.3
**Household wealth index score (Mean ± SD)** (*n*** = 191**)	
Lowest quintile (*n* = 35)	-2.2 ± 0.3
Lowest middle quintile (*n* = 38)	-0.8 ± 0.3
Lower middle quintile (*n* = 38)	-0.02 ± 0.1
Upper middle quintile (*n* = 41)	0.5 ± 0.2
Upper most quintile (*n* = 39)	2.4 ± 1.0

*n*(%) represents Frequency (Percentage). *Family type has been operationally defined as (a) Nuclear family: Household with a married couple and their children; (b) Joint family: Household with multiple generations including grandparents, parents, and their children; (c) Extended family: Household with aunts, uncles, cousins, and grandparents

### Food environment

The Munda HHs accessed various indigenous (*n* = 193) as well as non-indigenous foods (*n* = 68) from both natural and built food environments, across monsoon and winter seasons. All HHs were accessing markets while about 70% of them were accessing various natural sources such as foods produced from own agricultural fields (92.5%), collected from forests (72%) and water bodies likes ponds and rivers (73%) (Table 1). Additionally, the community also reported consuming traditional as well as market-procured alcohol (*n* = 6), and freshly prepared meals, and ultra-processed food items (*n* = 16). As reported in free-listing enquiries, farms and kitchen gardens were mainly utilized for cultivation of indigenous as well as non-indigenous food items (30%) including different varieties of rice, pulses, and other vegetables. On the other hand, around 55% IFs like wild fruits, tubers, mushrooms, GLVs and wild game meat were seasonally foraged from forests, open spaces and water bodies. The HHs mainly accessed local markets for procuring more than one-fourth of their food items (29%), including non-indigenous varieties of rice, pulses, fruits, vegetables, flesh foods, cooking oil, nuts and seeds as well as specific varieties of indigenous GLVs and other vegetables. At the same time, markets also served as key focal point for purchase of freshly prepared snacks and savories, and ultra-processed foods like chips, biscuits and namkeen, constituting 5.6% of the main foods consumed by Munda community. Details of specific food items in monsoon and winter seasons are provided in Supplementary file 2. .

### Dietary patterns

**Fig. 2:**
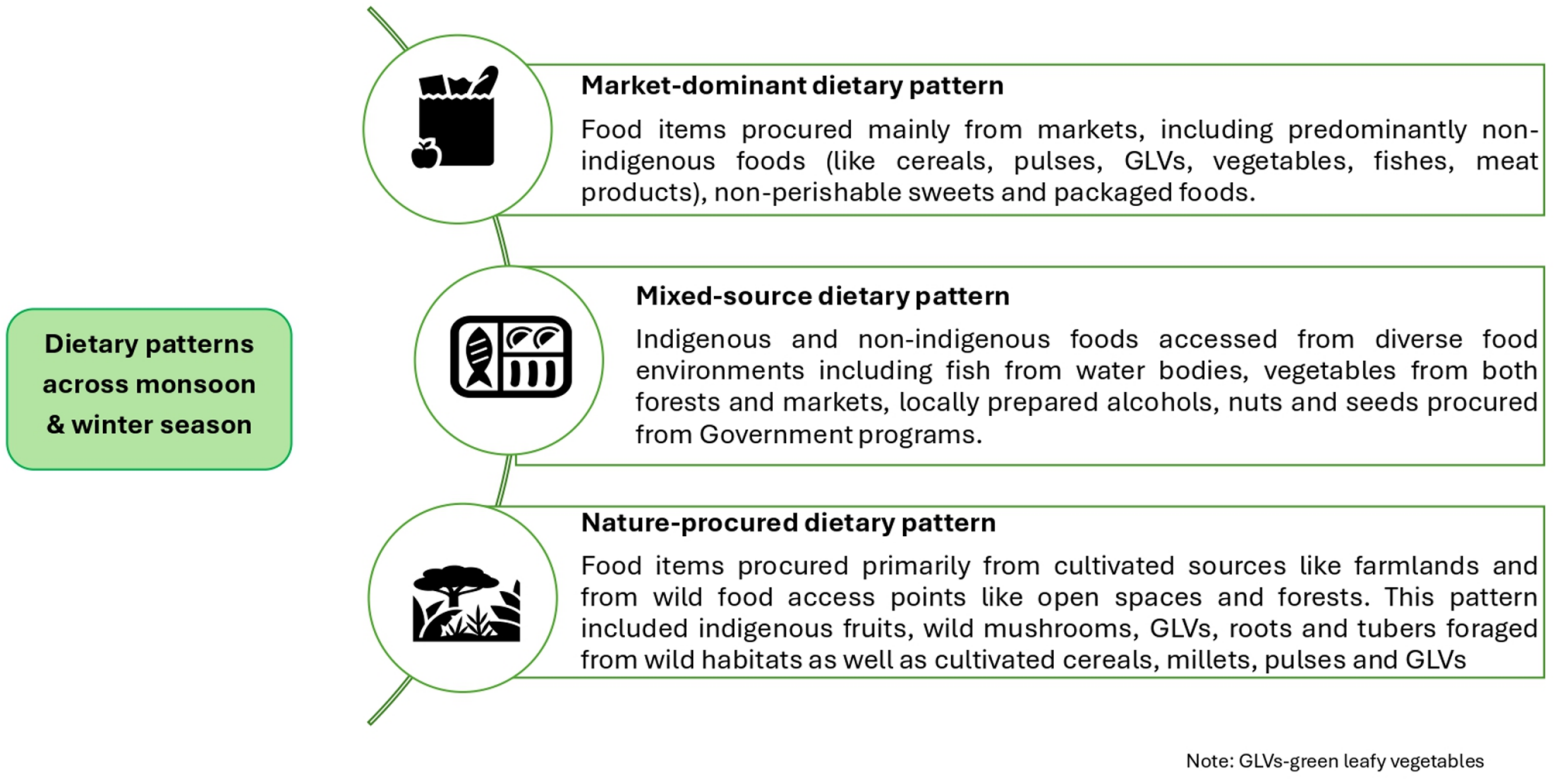
Dietary patterns identified for monsoon and winter season after factor analysis

In the Munda indigenous community, multiple dietary patterns were observed for the two seasons (Fig. 2). These dietary patterns were identified by applying the PCA statistical method. After applying the PCA, three factors were extracted for both monsoon and winter seasons. The first three factors in each season were given the name of a dietary pattern as shown in Tables 2 and 3. These tables also show the list of food sub-categories that predominantly appeared in the patterns along with the contribution of loadings to each of the factors.

**Table 2:** Factor loadings of 64 food sub-categories in principal component analysis of FFQ for monsoon season (*n* = 160)

Food categories	Market-dominant dietary pattern	Nature-procured dietary pattern	Mixed-source dietary pattern
Commonly consumed non-indigenous cereal staples	-0.034	-0.145	0.191
Less-commonly consumed non-indigenous cereals	0.102	0.434	0.264
Non-indigenous millets	0.079	0.483	0.385
Non-indigenous cereals eaten as a snack	0.419	0.133	-0.055
Commonly consumed indigenous cereal staples	0.124	0.324	-0.258
Less-commonly consumed indigenous cereals	-0.045	0.362	0.247
Indigenous millets	-0.143	0.284	0.198
Commonly consumed non-indigenous pulses	0.191	-0.008	0.462
Less commonly consumed non-indigenous pulses	0.492	0.007	0.433
Commonly consumed indigenous pulses	-0.085	0.116	0.189
Less commonly consumed indigenous pulses	0.049	0.354	-0.601
Milk and milk products from the market	0.036	-0.03	-0.28
Non-indigenous meat products from reared livestock	0.481	-0.077	0.281
Non-indigenous meat products from market	0.641	-0.197	-0.225
Indigenous meat products from forest/field	0.613	-0.221	0.075
Indigenous meat products from reared livestock	0.168	0.074	-0.13
Non-indigenous fish	0.352	0.018	-0.102
Non-indigenous molluscs	0.239	0.001	-0.062
Commonly consumed indigenous fish from fishing	0.568	-0.112	-0.027
Less commonly consumed indigenous fish from fishing	0.15	0.071	-0.025
Commonly consumed indigenous molluscs from fishing	0.115	0.066	0.008
Non-indigenous fruits from roadside and wasteland	-0.423	-0.039	-0.125
Non-indigenous fruits from market	0.607	0.096	0.341
Indigenous fruits from forests and/or open spaces	0.022	0.468	-0.148
Non-indigenous GLVs commonly consumed from market	0.331	-0.356	-0.005
Non-indigenous GLVs consumed as condiments/herbs	0.644	0.008	-0.024
Commonly consumed indigenous GLVs obtained from forest	0.223	-0.064	-0.146
Less commonly consumed indigenous GLVs obtained from forest	0.123	0.09	-0.069
Commonly consumed indigenous GLVs growing as weeds	0.129	0.315	-0.176
Less-commonly consumed indigenous GLVs growing as weeds	0.206	0.423	-0.054
Commonly consumed indigenous GLVs cultivated	0.002	0.043	-0.302
Less commonly consumed indigenous GLVs cultivated	0.536	0.136	0.054
Non-indigenous vegetables from market	0.568	-0.098	0.344
Non-indigenous vegetables cultivated	0.055	-0.003	0.461
Non-indigenous vegetables from open spaces	-0.008	-0.158	-0.038
Less commonly consumed indigenous vegetables from forests	0.665	0.036	0.318
Commonly consumed indigenous vegetables from market	0.192	0.299	-0.094
Commonly consumed indigenous vegetables cultivated	-0.03	-0.01	-0.088
Less commonly consumed indigenous vegetables cultivated	0.121	0.218	0.203
Commonly consumed mushrooms	0.16	-0.284	0.105
Less-commonly consumed mushrooms	0.194	0.39	-0.183
Non-indigenous roots and tubers as a vegetable preparation from market	-0.408	-0.003	0.153
Non-indigenous roots and tubers used as a condiment from market	-0.066	-0.039	-0.297
Non-indigenous roots and tubers cultivated	-0.026	0.541	0.233
Commonly consumed indigenous tubers from forest	-0.041	0.306	0.086
Less commonly consumed indigenous tubers from forest	0.017	0.128	-0.029
Commonly consumed indigenous tubers cultivated	0.001	0.168	-0.027
Non-indigenous saturated oils and fats from market	0.05	-0.066	-0.369
Non-indigenous unsaturated oils from market	0.102	0.413	-0.034
Non-indigenous unsaturated oils derived from pressing seeds	0.017	-0.379	-0.042
Home produced indigenous oils and fats	0.01	0.148	-0.149
Nuts and oilseeds from Govt. programme	-0.035	0.149	0.014
Nuts and oilseeds from market	0.021	0.298	-0.066
Sugars from forest	0.041	0.24	0.029
Sugars from market	0.515	0.015	0.3
Sugars from TPDS	0.357	-0.272	0.306
Alcohols prepared locally	0.187	-0.467	0.056
Alcohols bought from the market	0.158	0.099	0.118
Miscellaneous foods eaten as a side dish	0.459	-0.16	0.136
Cooked Sweets and desserts	0.179	0.173	-0.432
Freshly prepared dishes from the market	0.261	0.108	-0.031
Packaged foods	0.39	-0.385	0.021
Hot beverage	0.342	-0.273	0.252
Non-perishable sweets	0.33	0.138	-0.064

GLV-Green leafy vegetable; TPDS-Targeted Public Distribution System; factor loadings of ≥|0.3|have been considered to define a dietary pattern

Indigenous foods or IFs: species of plant/animal foods native to the place that are accessed through both natural and built food environment and are culturally acceptable; non-indigenous foods: species of plant/animal foods that are not native to the place and have been introduced through human activity, either deliberately or accidentally. These foods are mainly procured through markets, but may also be grown in fields and kitchen gardens [36, 43]

**Table 3:** Factor loadings of 60 food sub-categories in principal component analysis of FFQ for winter season (*n* = 100)

Food categories	Nature-procured dietary pattern	Mixed-source dietary pattern	Market-dominant dietarypattern
Commonly consumed non-indigenous cereal staples	0.081	0.053	0.042
Less-commonly consumed non-indigenous cereals	0.066	-0.008	0.165
Non-indigenous millets	0.339	0.014	0.129
Non-indigenous cereals eaten as a snack	0.008	0.156	0.5
Commonly consumed indigenous cereal staples	0.143	-0.036	0.302
Less-commonly consumed indigenous cereals	-0.088	-0.1	-0.075
Indigenous millets	-0.03	0.233	-0.086
Commonly consumed non-indigenous pulses	0.235	0.349	-0.177
Less commonly consumed non-indigenous pulses	0.037	-0.036	0.212
Commonly consumed indigenous pulses	0.198	-0.1	0.26
Less commonly consumed indigenous pulses	-0.045	0.144	0.168
Milk and milk products from the market	0.379	0.237	0.134
Non-indigenous meat products from reared livestock	0.059	0.059	0.325
Non-indigenous meat products from market	-0.025	0.017	0.357
Indigenous meat products from forest/field/open spaces	0.503	0.107	-0.102
Non-indigenous fish	-0.026	-0.08	0.18
Commonly consumed indigenous fish from water bodies	0.012	0.309	0.166
Less commonly consumed indigenous fish from water bodies	0.01	0.034	-0.02
Commonly consumed indigenous molluscs from water bodies	0.017	0.069	-0.087
Non-indigenous fruits from open spaces	0.02	0.337	0.102
Non-indigenous fruits from market	0.053	-0.084	0.367
Indigenous fruits from forests and/or open spaces	-0.003	0.199	0.01
Commonly consumed non-indigenous GLVs cultivated	0.283	-0.059	0.525
Commonly consumed non-indigenous GLVs from market	0.143	0.03	0.135
Non-indigenous GLVs consumed as condiments/herbs	0.027	-0.061	0.411
Commonly consumed indigenous GLVs obtained from forest	0.059	-0.084	0.063
Less commonly consumed indigenous GLVs obtained from forest	0.004	0.369	0.141
Commonly consumed indigenous GLVs growing as weeds	0.063	-0.338	0.113
Commonly consumed indigenous GLVs cultivated	0.204	0.178	0.306
Less commonly consumed indigenous GLVs cultivated	0.561	0.187	-0.17
Non-indigenous vegetables from market	0.106	0.556	0.079
Non-indigenous vegetables cultivated	0.068	0.304	0.206
Less commonly consumed indigenous vegetables from forests	0.792	0.133	0.037
Commonly consumed indigenous vegetables from market	0.456	0.492	-0.058
Commonly consumed indigenous vegetables cultivated	0.286	0.429	-0.188
Less commonly consumed indigenous vegetables cultivated	0.015	-0.005	0.04
Non-indigenous roots and tubers as a vegetable preparation from market	-0.264	0.552	-0.502
Non-indigenous roots and tubers used as a condiment from market	-0.123	0.356	0.378
Non-indigenous roots and tubers cultivated	0.04	0.126	0.059
Commonly consumed indigenous tubers from forest	-0.127	0.065	-0.061
Less commonly consumed indigenous tubers from forest	-0.086	0.168	0.126
Commonly consumed indigenous tubers cultivated	-0.093	0.234	0.027
Less commonly consumed indigenous tubers cultivated	-0.084	0.026	0.006
Non-indigenous saturated oils and fats from market	0.036	-0.287	0.039
Non-indigenous unsaturated oils from market	0.003	-0.081	0.418
Non-indigenous unsaturated oils derived from pressing seeds	-0.172	0.224	0.092
Home produced indigenous oils and fats	0.15	-0.108	-0.168
Nuts and oilseeds from Govt. programme	0.08	0.625	-0.086
Nuts and oilseeds from market	-0.04	0.019	0.045
Sugars from forest	0.84	-0.07	-0.042
Sugars from market	-0.084	0.148	0.427
Sugars from TPDS	0.124	0.452	0.521
Alcohols prepared locally	-0.157	0.16	0
Alcohols bought from the market	-0.091	0.157	0.296
Miscellaneous foods eaten as a side dish	0.129	0.245	0.044
Cooked sweets and desserts	-0.045	0.068	0.223
Freshly prepared dishes from the market	0.503	-0.032	0.161
Packaged foods	0.007	0.056	0.413
Hot beverage	0.077	0.471	0.538
Non-perishable sweets	-0.097	0.146	0.301

GLV-Green leafy vegetable; TPDS-Targeted Public Distribution System; factor loadings of ≥|0.3|have been considered to define a dietary pattern

Indigenous foods or IFs: species of plant/animal foods native to the place that are accessed through both natural and built food environment and are culturally acceptable; non-indigenous foods: species of plant/animal foods that are not native to the place and have been introduced through human activity, either deliberately or accidentally. These foods are mainly procured through markets, but may also be grown in fields and kitchen gardens [36, 43]

### Monsoon season

The FFQ data from 160 HHs was used to run the factor analysis. The factors derived from PCA contributed some variance to the dataset. This variance explains how much each factor explains a particular dataset. For the monsoon season, three dietary patterns were identified which together explained 19.7% of dietary intake of the Munda community. The first dietary pattern was categorized as ‘market-dominant pattern’. This dietary pattern mainly included food items purchased from the market, including non-indigenous cereals eaten as snacks (rice flakes or puffed rice), non-indigenous pulses, non-indigenous GLVs, non-indigenous vegetables, non-perishable sweets and packaged foods This pattern also included indigenous and non-indigenous meat products sourced from markets, forests, fields, or through reared livestock. Indigenous as well as non-indigenous fishes, accessed from both markets and water bodies, also formed an important component of this dietary pattern.

The second dietary pattern labelled as a “nature-procured dietary pattern’ predominantly comprised food items procured from cultivated and wild food environments. This dietary pattern was characterized by foods like indigenous fruits accessed from forests and/or open spaces, indigenous GLVs foraged from open spaces and cultivated fields, wild mushrooms as well as indigenous and non-indigenous roots and tubers collected from forests. Cultivated as well as market-procured indigenous and non-indigenous cereals and millets, and indigenous pulses also contributed to this pattern.

The third dietary pattern was labelled as ‘mixed-source dietary pattern’ as it mainly consisted of indigenous and non-indigenous food items obtained from different sources like forests, cultivated farmlands, markets and government supplementary feeding programs. This dietary pattern included non-indigenous millets, indigenous and non-indigenous pulses, non-indigenous fruits, non-indigenous vegetables and sugars.

### Winter season

Table 3 shows the factor loadings for three components of the winter season (*n* = 100 HHs). These three factors or principal components explained 17.2% of the total variance in food intake. Individually, the first factor explained 5.5% variance, second factor explained 5.7% variance and the third factor explained 6% variance.

The first emerging dietary pattern categorized as ‘nature-procured dietary pattern’ had factor loadings ≥ 0.3 for food items like indigenous meat products from forests and open spaces, cultivated GLVs, indigenous vegetables and honey obtained from forests.

The second dietary pattern was labelled as ‘mixed-source dietary pattern’ because it had positive factor loadings (≥ 0.3) from multiple food groups accessed from various food environments. These included non-indigenous pulses, indigenous fish obtained from water bodies, indigenous GLVs obtained from forest, indigenous fruits purchased from the market, market-procured as well as cultivated varieties of indigenous and non-indigenous vegetables, nuts and sugars obtained from Government supplementary feeding programmes, and hot beverages like tea.

The third dietary pattern was labelled as a ‘market-dominant dietary pattern’ as it predominantly comprised different foods procured from the market with factor loadings ≥ 0.3. This dietary pattern included indigenous as well as non-indigenous cereals eaten as snacks (like puffed rice and rice flakes), non-indigenous varieties of meat products, fruits, GLVs, and roots and tubers, procured from the market. Additionally, cooking oil, sugars, alcohols and packaged branded and unbranded foods (like chips, biscuits, and chocolates) also formed an important component of this dietary pattern.

### Factors associated with dietary patterns

Logistic regression was carried out to assess the association between dietary pattern scores with various socio-demographic and food environment factors. For the monsoon season, it was found that HHs with higher FADI scores had 3.93 times the odds of consuming more “mixed-source dietary pattern” (OR-3.93, *p*-value = 0.034) (Supplementary file 4). Additionally, HHs practicing settled agriculture as their primary occupation were more likely to consume more of “market-dominant dietary pattern” (OR = 2.09, *p*-value = 0.048), in comparison to HHs with other primary sources of income. For the winter season, families living in a nuclear family setup had significantly higher odds of consuming more market-dominant (OR = 2.90, *p*-value = 0.008) and nature-procured dietary patterns (OR = 2.62, *p*-value = 0.016), as compared to families living in joint/extended family setup (Supplementary file 5).

## Discussion

In the present study, the dietary patterns of Munda indigenous community were explored for two seasons - monsoon and winter. After analysing results from the PCA, three distinct dietary patterns were observed in each of the two seasons: nature-procured pattern, market-dominant dietary pattern and mixed-source dietary pattern. These specific patterns (a score symbolizing each of the distinct dietary patterns) were further associated with sociodemographic and food environment factors at the HH level. Food access diversity index (FADI), family structure, and agricultural land access were associated with specific patterns.

A mixed-source dietary pattern was observed in both seasons, which included foods procured from both wild and built food environments. Our previous studies on food environment profiling of the Munda community have demonstrated access to both the natural food environment and markets in the study villages [43]. The markets accessed by the Munda community have a range of both nutrient-dense foods (like vegetables grown in kitchen gardens, green leafy vegetables, tubers, and fruits sourced from wild habitats) and a range of unhealthy packaged foods that are high in salt, sugar, and fat with attractive packaging [43]. The market-accessed foods in our mixed pattern were majorly packaged foods, while the community also relied on a natural food environment for freshly grown foods like cereals, green leafy vegetables, and other vegetables. Further, the market-dominant dietary pattern in Mundas, which included a good proportion of packaged foods procured from the markets, was a reflection of the phenomenon of nutrition transition among the community, whose food procurement practices are changing. Similar findings are reported in other indigenous communities of India and across the globe, who are integrating foods considered to be unhealthy, such as packaged ultra-processed foods, fast food, and junk foods into their traditional diets [43, 45, 53]. These studies further demonstrate an association between HH income and consumption of unhealthy foods, with low-income populations consuming unhealthy foods more frequently [53–55]. Routine consumption of such diets may also place the communities at risk of diet-related non-communicable diseases. For example, studies among rural and peri-urban populations in Africa have shown a link between greater consumption of processed and ultra-processed foods, with increasing prevalence of obesity, higher HbA1c levels and other NCD risk factors [56, 57]. The study findings further highlighted that nuclear families were more likely to have market-dominant dietary patterns, indicating that families with lesser family members tend to rely on market-purchased foods, thus influencing their food choices [58].

The increased access to markets is leading to the consumption of both healthy and unhealthy foods among indigenous communities. In our study, families with settled agriculture had higher likelihood of consuming market-dominant dietary patterns. A possible reason for this association could be attributed to limited crop yields, thus leading to reduced dependence on home-grown foods, steering a shift towards easy-to-procure market foods. However, it is important to understand that markets can also be the focal access points of procuring nutrient-rich IFs that can potentially address malnutrition in indigenous communities. This may also lead to an indirect positive influence on the livelihood of the villagers, smallholder farmers, and forest dwellers who sell IFs in local markets. A study in the provinces of Limpopo and Mpumalanga in South Africa highlighted improved HH food security of smallholder farmers who partook in the marketing of indigenous crops [59]. Hence, there is a need for providing information education and communication to the indigenous communities regarding the identification of healthier options from markets [43, 45].

Further, it is increasingly recognized that owing to the cultural and social norms of indigenous communities, engaging them in supplying IFs to the market is an environmentally and economically sustainable approach [60]. Hence, promoting a consumption behavior that prioritizes accessing nutritious IFs from the market while reducing the opportunity cost of foraging from natural resources could be a crucial strategy for tweaking the existing dietary patterns of Munda community and making it healthier for them.

The Munda community also demonstrated access to diverse IF sources [42, 44]. This was also reflected in the distinct nature-procured dietary pattern reported in our study, across both monsoon and winter seasons. This pattern highlighted the consumption of IF items like meats, green leafy vegetables and honey which were procured from cultivated and wild food environments. Other studies have demonstrated similar dietary patterns referred to as ‘traditional dietary pattern’ that showed high factor loadings for cereals and grains, local beverages, nuts, seeds and legumes, vegetables, and fish and seafood among different populations of adolescents, adults and indigenous communities [61, 62]. FAO has reiterated the importance of indigenous knowledge for sustainable transformations of food system. However, this strategy often remains marginalized in policy and practice [63]. The observed dietary pattern in our study would need reinforcement by identifying key strategies to successfully utilize IF systems. Among the Khasi tribal communities of India, with support from an Indigenous Peoples’ non-profit organization, the communities are working to increase the production and consumption of local micronutrient-rich and climate-resilient species. Various activities, including establishing community seed banks and community gardens, and promoting resilient agroecological practices, have been reported as part of this initiative [36].

## Conclusion

In the present study, different dietary patterns comprising a mix of indigenous and non-indigenous foods procured from both natural food access points and markets were observed. These dietary patterns were closely associated with specific food environment and socio-demographic factors. The dietary patterns highlighting access to processed and ultra-processed foods from the markets indicate the phenomenon of nutrition transition that is evident in resource-poor rural and tribal communities of India. This transition can perhaps be tilted toward accessing markets that act as a source of acquiring both commercial foods as well as traditional IFs that are potentially healthier options. This, however, would require undertaking health and nutrition promotion activities to develop contextual messages on accessing markets for purchasing nutrient-rich naturally produced IFs with a shorter supply chain and sold by local vendors. Identifying the dietary patterns is also critical to inform the current policies and programs trying to mainstream indigenous wisdom and knowledge to improve the diets of nutritionally vulnerable indigenous communities. The information on prominent patterns that continue to utilize IFs is reassuring and community-based food supplementation programs can be restructured to promote the beneficial dietary patterns for better uptake in the community. Finally, this evidence-supported information on prominent dietary patterns can facilitate understanding the level of nutrition and food environment transition in indigenous communities. It can provide objective indicators for influencing policies and guidelines around commercial determinants of health and nutritional well-being in these populations.

## Supplementary Information

Below is the link to the electronic supplementary material.

Supplementary Material 1

Supplementary Material 2

Supplementary Material 3

Supplementary Material 4

Supplementary Material 5

## Data Availability

The datasets used and analysed during the current study are available from the corresponding author on reasonable request.

## Abbreviations

CAPI: Computer-asssisted personal interviewing
FADI: Food access diversity index
FAO: Food and agricultural organisation
FFQ: Food frequency questionnaire
GLV: Green-leafy vegetables
HH: Household
IF: Indigenous food
LMIC: Low and middle-income countries
NCD: Non-communicable diseases
PCA: Principal component analysis
ST: Scheduled tribes
TEK: Traditional ecological knowledge
TPDS: Targeted public distribution system

## Footnotes

1 The term “Indigenous foods or IFs” in our study refers to foods that are native or have been introduced in the region a long time back and are accessed from natural food environment (i.e. forests, rivers, ponds, lakes, farms, kitchen gardens, wastelands, pastures and roadsides). Considering the globalization of current food systems, these foods may also be purchased from informal built food environment i.e. local markets or small shops [36, 52].
