# Supplementary material for: Indigenous food environment and dietary patterns of Munda community of Jharkhand, India
Source: BMC Nutr. 2025 Oct 21;11:189. doi: 10.1186/s40795-025-01159-2 (PMC12539013; doi:10.1186/s40795-025-01159-2)
Supplement: Supplementary file 1 — Supplementary Material 1 [file 40795_2025_1159_MOESM1_ESM.pdf]

DATE | | | | | | | | |

GPS coordinates | |

Household Survey Questionnaire

INSTRUCTIONS: This questionnaire contains 4 parts and will be administered to any adult member (preferably female and married) of the household.

File opens in the EDC form

Household ID: | | | | | | | | | | | |

Has consent been taken?	1 ☐ Yes 2 ☐ No

Section 1: Background Information

Q No.	Question	Response	Comments
1.	Name of the Respondent		
2.	Gender	1. Male<br>2. Female	
3.	Religion	1. Hindu<br>2. Muslim<br>3. Christian<br>96. Any Other (Please Specify) ___________	
4.	Type of Family	1. Nuclear<br>2. Joint<br>3. Extended	
5.	Educational Status of Head of Household	Common coding for Q 5,6 and 7<br>1.No formal education<br>2. No formal education but can sign<br>3.No formal education but can read and write<br>4. Less than primary (5th class)<br>5. Completed Primary<br>6.More than primary (5th class) but less than secondary (10 th class)<br>7.More than secondary but less than HS (12th class)<br>8.HS (12th class) and above	
6.	Educational Status of Participant 1<br>Check: Is [P1 name], the mother of [P2 name]?		If yes, skip to question 8
7.	Educational Status of Mother of Index child/ Participant 2		

8.	Occupation of Head of the Household in the current season	Common coding for Q 8, 9 and 10<br>1.Hunting/Gathering<br>2.Shifting Cultivation<br>3.Settled Agriculture<br>4. Craftsmen/ Artisans<br>5.Service (Govt. or Private)/Regular Salary<br>6.Daily wager (Construction Labour/Coolie/agriculture labour)<br>7.Self-employed (Tailor/Shop-owner)<br>8.Unemployed<br>9. Housewife<br>96.Other	
9.	Occupation of Participant 1 (selected woman) in the current season.		
10.	Occupation of Mother of Index Child/Participant 2 in the current season.		
11.	Type of House	1 Pakka<br>2. Semi-Pakka<br>3. Kaccha	
12.	Ownership of the house in which family is currently living?	1. Self owned<br>2. Rented	
13.	Beside the house in which family is living, do you own any other house?	1. Owns 2 or more<br>2. Owns 1<br>3. Doesn't own any	
14.	Number of Rooms in Household ?		
15.	Is there a separate kitchen in the house?	1.Yes<br>2.No	
16.	Type of Kitchenware being used at home? (Multiple response)	16.1 Clay/Earthen A<br>16.2 Aluminium B<br>16.3 Cast iron C<br>16.4 Stainless Steel D<br>16.5 Copper/Brass E	Read out all the options. Select all the options that are applicable
17	Which are the two major sources contributing towards food items included in your daily diet in the current season?	**i……………………………..**<br><br>**ii…………………………….**<br><br>**Options for i and ii**<br>1. Agricultural produce<br>2. Bari<br>3. Forest/ common space<br>4. Bought from Market<br>5. From Exchange<br>6. PDS (ration shop)	

18	Which is the source that contributes least towards the food items present in your daily diet in the current season?	1. Agricultural produce<br>2. Bari<br>3. Forest/ common space<br>4. Bought from Market<br>5. From Exchange<br>6. PDS (ration shop)	
19	Which ration card do you possess?	1. Green<br>2. Red<br>3. Yellow<br>4. No ration card	If response is 4, skip to Q 24
20	How much amount of the following do you get from the ration shop per month?		(Use the local name for PDS shop)
	23.1 Rice (kg)		
	23.2 Salt (kg)		
	23.3 Sugar (kg)		
	23.4 Kerosene Oil (ltr)		
	23.5 Any Other<br>(Please Specify)		
21.	How much do you spend approximately on food items per month (rice, pulse, oil, sugar, etc.)?	Rupees____________________	Ask respondent on weekly basis how much is spent and then multiply by 4.
22.	What is the predominant source of fuel in your house?	1. Electricity 2. LPG<br>3. Kerosene 4. Gobar gas/Biogas<br>5. Coke,Coal 6.Charcoal<br>7.Dungcakes<br>8.Firewood/chips/grass/stems/straw/shrub/agriculture waste<br>96. Any Other (…………………………..)	
23.	What is the predominant source of light in your house?	1. Electricity 2. Solar Panels<br>3. Kerosene 4. Any other oil<br>5.Gas<br>6. Bio gas/ Gobar gas 7. Candle<br>8. None	

24.	What is the main source of drinking water?	1.Piped water<br>2.Well<br>3.Tank/Pond<br>4. River/Dam/spring /waterfall<br>5 Tube well/Hand-pump<br>96. Any other (Please Specify)<br>………………………	
25.	Where do the adults in the family go for defecation?	1. Own Toilet<br>2. Shared Toilet (with other HH)<br>3. Public Toilet (Community toilet)<br>4. Open Field/ Jungle	
26.	Do you have any outstanding debt?	1. Yes<br>2. No	If response is 2, skip to Q.31
27.	Reason for the Debt	1. Purchasing new land<br>2. Expenditure on Agriculture<br>3. Expenditure on Manufacture<br>4. Marriage/Death/Birth Ceremony<br>5. Other Household Expenditure<br>6. Repayment of old Debts<br>7. Due to drinking<br>8. Gambling<br>9. Health expenditure<br>96. Others	
28.	What is distance to nearest market, from where food items are bought?	1.<1km<br>2.1-3Kms<br>3.3-5Kms<br>4.5-10 Kms<br>5. >10 kms	
29.	How do you access nearest market for your daily food needs?	1.Public Transport (Bus/Shared Auto)<br>2.Personal Transport (Bicycle/Bike)<br>3.Walking<br>4.Fetched by someone else<br>5. Don't go to market	
30	Do you collect food items from forest?	1. Yes 2. No	
31	What is distance to nearest forest, from where food items are bought?	1.<1km<br>2.1-3Kms<br>3.3-5Kms<br>4.>5 Kms	

Section 2: Agriculture and Livestock Information

S No.	Question	Response	Comment

33	Do you engage in agricultural practices in the current season?	1. Yes, in own land<br>2. Yes, on leased land<br>3. Kurwa<br>4. Own land & Kurwa<br>5. Leased land & Kurwa<br>6. None		If response is 3, skip to Q 35
34	Do you grow crops in Goda/Taar?	1. Yes 2. No		
34	What are different crops grown?	A.<br>1.Yes<br>2.No	B. Major Use:<br>1.Household Consumption<br>2. Sold to market<br>3. Both Household consumption and sold in market (equal amounts)<br>3.Exchanged for other goods/services<br>4.Gift to neighbours<br>96.Others (Specify)	
	34.1 Pundigoda			
	34.2 Jondra/Gangayi/Makka (maize)			
	34.3 Bajra /Gangayi			
	34.4.Ragi/Mandua			
	34.5.Kulthi			
	34.6 Rehad			
	34.7 Urad/Rambada Dal			
	34.8 Kanau			
	34.9 Jojoarak			
	34.10 Sanai Phool			
	34.11 Shakarkand			
	34.12 Sambalpuri rice			
	34.13 Hengdehgoda rice			
	34.14 Haselsar rice			
	34.15 Pundi baba			
	34.16 Chorayagoda rice			

	34.17 Safed Dhan			
	34.18 Lal Dhan			
	34.19 Any other crop_1 (Specify……………………….)			
	34.20 Any other crop_2 (Specify………………………)			
	34.21 Any other crop_3 (Specify……………………..)			
	34.22 Any other crop_4 (Specify……………………..)			
	34.23 Any other crop_5 (Specify…………………….)			
	34.24 Any other _crop_6 (Specify…………………….)			
35	Do you grow crops in Badi/Chora?	1. Yes<br>2. No		
	What are different crops grown?	A.<br>1.Yes<br>2.No	B. Major Use:<br>1.Household Consumption<br>2. Sold to market<br>3. Both Household consumption and sold in market (equal amounts)<br>3.Exchanged for other goods/services<br>4.Gift to neighbours<br>96.Others (Specify)	
	35.1 Chota Ghangra			
	35.2 Potato			
	35.3 Sarson saag/Mani ada			
	35.4 Jondra/Gondli/Makai			
	35.5 Onion			
	35.6 Tomato			
	35.7 Brinjal			
	35.8 Jheenga			
	35.9 Murai			
	35.10 Any other crop_1 (Specify……………………….)			
	35.11 Any other crop_2 (Specify……………………….)			

	35.12 Any other crop_3 (Specify……………………….)			
	35. 13 Any other crop_4 (Specify……………………….)			
	35.14 Any other crop_5 (Specify……………………….)			
	35.15 Any other crop_6 (Specify……………………….)			
36	Do you grow crops in Loyong/Don?	1. Yes 2. No		
36	What are different crops grown?	A.<br>1.Yes<br>2.No	B. Major Use:<br>1.Household Consumption<br>2. Sold to market<br>3. Both Household consumption and sold in market (equal amounts)<br>3.Exchanged for other goods/services<br>4.Gift to neighbours<br>96.Others (Specify)	
	36.1 Gitilbaba			
	36.2 Arababa			
	36.3 Jolpo dhan			
	36.4 Dhusri dhan			
	36.5 Jhili			
	36.6 Budha dhan/Haran baba			
	36.7 Nabin			
	36.8 Payonir baba			
	36.9 Duru baba			
	36.10 Shankar dhan			
	36.11 Any other crop_1 (Specify……………………….)			
	36.12 Any other crop_2 (Specify……………………….)			
	36.13 Any other crop_3 (Specify……………………….)			
	36. 14 Any other crop_4 (Specify……………………….)			

	36.15 Any other crop_5 (Specify……………………….)			
	36.16 Any other crop_6 (Specify……………………….)			
37	Do you grow any vegetable/ crops/fruit/ food items in the backyard or in the empty space around your home (Baari) in the current season?	1. Yes<br>2. No		If response is 2, skip to Q 37
37	What vegetables/crops are grown in the Baari? Food items grown in the Baari (vegetables/crops/ fruits)	A.<br>1.Yes<br>2. No	B. Major Use<br>1.Household Consumption<br>2. Sold to market<br>3. Both Household consumption and sold in market (equal amounts)<br>3.Exchanged for other goods/services<br>4.Gift to neighbours<br>96.Others (Specify)	
	37.1 Makka			
	37.2 Sarson saag/Mani ara			
	37.3 Onion			
	37.4 Tomato			
	37.5 Brinjal			
	37.6 Potato			
	37.7 Murai			
	37.8 Leped ara			
	37.9 Kakdu ara			
	37.10 Saru ara			
	37.11 Any other crop_1 (Specify……………………………….)			
	37.12 Any other Crop_2 (Specify……………………………….)			
	37.13 Any other Crop_3 (Specify……………………………….)			
	37.14 Any other Crop_4 (Specify……………………………….)			
	37.15 Any other Crop_5 (Specify……………………………….)			
	37.16 Any other Crop_6 (Specify……………………………….)			

37A	Was any weed grown in Goda/Bari/Loyong or Bakdi, collected?	1. Yes 2. No		
37A	What different foods were collected?	A.<br>1.Yes<br>2.<br>No	B. Major Use<br>1.Household Consumption<br>2. Sold to market<br>3. Both Household consumption and sold in market (equal amounts)<br>3.Exchanged for other goods/services<br>4.Gift to neighbours<br>96.Others (Specify)	
	1 Choke-ara			
	2 Sirgiti-ara			
	3 Uli-ara			
	4 Pasodababa			
	5 Garondi-ada			
	6 Minjri dhan			
	7 Teeri-reeti ara			
	8 Leped ara			
	9 Bathua saag			
	10 Muchri saag			
	11 Baji saag			
	12 Charmani saag			
	13 Kecho ara			
	14 Lundi ara			
	15 Mui ara			
	16 Any other crop_1<br>(Specify……………………………….)			
	17 Any other Crop_2<br>(Specify……………………………….)			
	18 Any other Crop_3<br>(Specify……………………………….)			
	19 Any other Crop_4<br>(Specify……………………………….)			

	20 Any other Crop_5<br>(Specify……………………………….)			
	21 Any other Crop_6<br>(Specify……………………………….)			
38	Do you access pond/river/small streams in the current season for getting food for family?	1. Yes<br>2. No		If response is 2, skip to Q 39
38	What are animals from pond/river/small streams you have access to?	A.<br>1.Yes<br>2.No	B.Major Use:<br>1.Household Consumption<br>2. Sold to market<br>3. Both Household consumption and sold in market (equal amounts)<br>3.Exchanged for other goods/services<br>4.Gift to neighbours<br>96.Others (Specify)	
	38.1.Fishes			
	38.2 Snails			
	38.3 Turtle			
	38.4 Iccha/Chingri/Prawns			
	38.5 Any other_1<br>(Specify……………………………….)			
	38.6 Any other _2<br>(Specify……………………………….)			
	38.7 Any other_3<br>(Specify……………………………….)			
	38.8 Any other_4<br>(Specify……………………………….)			
	38.9 Any other_5<br>(Specify……………………………….)			
	38.10 Any other_6<br>(Specify……………………………….)			
39	Do you have access to forests in the current season?	1. Yes<br>2. No		If response is 2, skip to Q 41.
40	What are the items you collect from forest or space around your house?	A.<br>1.Yes<br>2. No	B. Major Use:<br>1.Household Consumption<br>2. Sold to market<br>3. Both Household consumption and sold	

			in market (equal amounts)<br>3.Exchanged for other goods/services<br>4.Gift to neighbours<br>96.Others (Specify)	
	40.1 Medicinal Herbs/Shrubs			
	40.2 Honey			
	40.3 Edible plants/ plant food items			
	40.4 Firewood			
	40.5 Wild animals for consumption			
	40.6 Mushrooms			
	40.7 Any other_1 (Specify……………………………….)			
	40.8 Any other_2 (Specify……………………………….)			
	40.9 Any other_3 (Specify……………………………….)			
	40.10 Any other_4 (Specify……………………………….)			
	40.11 Any other_5 (Specify……………………………….)			
	40.12 Any other_6 (Specify……………………………….)			
41	Do you possess/ share domestic animals?	1.Yes<br>2.No		If response is 2, skip to next section.

42	Name of the animal	A. Number	B. Major use<br>1.Household Consumption<br>2. Sold to market<br>3. Both Household consumption and sold in market (equal amounts)<br>3.Exchanged for other goods/services<br>4.Gift to neighbours<br>96.Others (Specify)	
	42.1 Cow			
	42.2 Buffalo			
		A. Number	B. Major use<br>1.Household Consumption<br>2. Sold to market	

			3. Both Household consumption and sold in market (equal amounts)<br>3.Exchanged for other goods/services<br>4.Gift to neighbours<br>96.Others (Specify)	
	42.3 Pig			
	42.4 Goat/Sheep			
	42.5 Hen/cock			
	42.6 Duck			
	42.7 Any other _1 (Specify………………………………)			
	42.8 Any other_2 (Specify………………………………)			
	42.9 Any other_3 (Specify………………………………)			
	42.10 Any other_4 (Specify………………………………)			
	42.11 Any other_5 (Specify………………………………)			
	42.12 Any other_6 (Specify………………………………)			
