# Supplementary material for: Indigenous food environment and dietary patterns of Munda community of Jharkhand, India
Source: BMC Nutr. 2025 Oct 21;11:189. doi: 10.1186/s40795-025-01159-2 (PMC12539013; doi:10.1186/s40795-025-01159-2)
Supplement: Supplementary file 2 — Supplementary Material 2 [file 40795_2025_1159_MOESM2_ESM.pdf]

Supplementary file 2

2a. Munda Food Frequency Questionnaire (Last 1 month) - Monsoon Season

Household ID: | | | | | | | | | | |

Date: | | | | | | |

Season ___________________________

S. No.	Food Items	Frequency of Intake	S. No.	Food Item	Frequency of Intake
**A**	**Cereals (Non-Indigenous)**		B.4	Daanidhan [दानीधान]	
A.1	Rice, Hybrid [चावल, हाईब्रिड]		B.5	Pasoda baba [पसडा बाबा]	
A.2	Guhum/ Wheat [गेंहू]		B.6	Karanga dhan [करांगा धान]	
A.3	Bajra/Gangayi [बाजरा/गंगाई]		B.7	Kannaudhan [कन्नौधान]	
A.4	Muri/ Puffed Rice [मूड़ी]		B.8	Raasdhan [रासधान]	
A.5	Corn/Makkai, Hybrid [मक्का/ मक्कई, हाईब्रिड]		B.9	Minjri [मिन्जरी]	
A.6	Rice Flakes/ chirwa [चिडवा]		B.10	Aara/Aarodhan [अरोधान]	
A.7	Whole wheat flour		B.11	Jolpo baba [जोल्पोबाबा]	
**B**	**Cereals (Indigenous)**		B.12	Gilti baba [गिलटीबाबा]	
B.1	Laaldhan/Arababa [लालधान/आराबाबा]		B.13	Pundi goda [पुंडी गोडा]	
B.2	Safeddhan [सफेदधान]		B.14	Rieci baba [राइसी बाबा]	
B.3	Jedengdhan [जेदेंगधान]		B.15	Karnidhan [कर्निधान]	

1=Every day (>2 times) 2=Every day (2 times) 3=Every day (1 time) 4=5 to 6 days/week 5=3 to 4 days /week 6=1 to 2 days/ week 7=Once in a fortnight 8=Once in a month 0=Never

S. No.	Food Items	Frequency of Intake	S. No.	Food Item	Frequency of Intake
B.16	Lalatdhan [ललाटधान]		**C**	**Pulses (Non-Indigenous)**	
B.17	Panchanwaye dhan [पचानवेधान]		C.1	Raher/Red gram (whole) [अरहर/रहरी/रहेड छिलके वाली]	
B.18	Dusridhan [दुसरिधान]		C.2	Raher/ Red gram (dehusked) [अरहर/रहरी/रहेड, बिना छिलके वाली]	
B.19	Hathipanjardhan [हाथीपंजरधान]		C.3	Kulthi [कुल्थी]	
B.20	Rajdhan [राजधान]		C.4	Masoor dal [मसूर]	
B.21	Sambalpuri [संबलपुरी]		C.5	Boot/ Bengal gram dal [बूट/चना]	
B.22	Garmi dhan/teba baba [गरमा धान/टेवाँ बाबा]		C.6	Moong/ green gram (whole) [मूंग, छिलके वाली]	
B.23	Pagla Goda [पगला गोड़ा]		C.7	Moong/green gram (dehusked) [मूंग, बिना छिलके वाली]	
B.24	Sorno baba [सोरनो बाबा]		C.8	Rambada dal/ Black gram/Urad (whole) [रामबडा छिलके वाली]	
B.25	Gudlu [गुड़लु]		C.9	Rambada dal/Black gram/Urad (dehusked) [रामबडा बिना छिलके वाली]	
B.26	Anthanawaye dhan [अनठानव्वे धान]		C.10	Rajma	
B.27	Bajra/Gangayi [बाजरा]		**D**	**Pulses (Indigenous)**	
B.28	Mandua/Kodhe/Janhe [मंडुआ/कोड़ेह/जन्हे]		D.1	Suthro [सुथरी]	
B.29	Mansuri		D.2	Kesari dal [केसरी दाल]	
B.30	Sankar dhaan		D.3	Baturi/Tiri riti [बाटूरी/तीरि रीती]	

1=Every day (>2 times) 2=Every day (2 times) 3=Every day (1 time) 4=5 to 6 days/week 5=3 to 4 days /week 6=1 to 2 days/ week 7=Once in a fortnight 8=Once in a month 0=Never

S. No.	Food Items	Frequency of Intake	S. No.	Food Item	Frequency of Intake
D.4	Danbudi/Ghangra [डंगबुडी/घांघरा]		E.5	Curd [दही]	
D.5	Raher/Red gram (whole) [अरहर/रहरी/रहेड छिलके वाली]		**F**	**Meat and Meat Products (Non-Indigenous)**	
D.6	Raher/ Red gram (dehusked) [रहेड, बिना छिलके वाली]		F.1	Meat/Mutton [मीट/बकरी/भेड़ का मांस]	
D.7	Boot/ Bengal gram dal [बूट/चना]		F.2	Beef [गाय का मांस]	
D.8	Masoor dal [मसूर]		F.3	Chicken [मुर्गा]	
D.9	Moong/ green gram (whole) [मूंग, छिलके वाली]		F.4	Eggs [अंडा]	
D.10	Moong/green gram (dehusked) [मूंग, बिना छिलके वाली]		F.5	Pig [सूअर]	
D.11	Kulthi [कुल्थी]		**G**	**Meat and Meat Products (Indigenous)**	
D.12	Rambada dal/Urad/Black gram (whole) [रामबडा छिलके वाली]		G.1	Jangli murgi [जंगली मुर्गी]	
D.13	Rambada dal/Urad/Black gram (dehusked) [रामबडा बिना छिलके वाली]		G.2	Rabbit [खरगोश/कुलय]	
D.14	Bodi		G.3	Jungli Peacock [जंगली मोर]	
**E**	**Milk and Milk products**		G.4	Tumbuli (insect)[तुमबुली]	
E.1	Cow's Milk [गाय का दूध]		G.5	Neelirasi [नीली रासी]	
E.2	Buffalo's Milk [भेंस का दूध]		G.6	Field Rat / Musa/Guddu/ [खेत का चूहा/मूसा/गुड्डु]	
E.3	Packaged Milk [पैकेट वाला दूध]		G.7	Jangli suar [बीर सुकरी / जंगली सूअर/ सिलीब]	
E.4	Paneer [पनीर]		G.8	Porcupine / Saahi/Jikki/Torod [किस्सा/साही/जिक्की/तोरोद]	

1=Every day (>2 times) 2=Every day (2 times) 3=Every day (1 time) 4=5 to 6 days/week 5=3 to 4 days /week 6=1 to 2 days/ week 7=Once in a fortnight 8=Once in a month 0=Never

S. No.	Food Items	Frequency of Intake	S. No.	Food Item	Frequency of Intake
G.9	Bando [बंडो]		G.25	Khasi [खासी]	
G.10	Hadhoga (Siyar)[हाधोगा/सीयार]		G.26	Demta [डेमटा]	
G.11	Pigeon (Kabutar)[कबूतर]		**H**	**Fish (Non-Indigenous)**	
G.12	Bees/ Madumakkhi [मधुमक्खी/मधु रस]		H.1	Katla [कतला]	
G.13	Chitri [चित्री]		H.2	Rohu [रोहू/रूही]	
G.14	Duhur [दुहुर]		H.3	Katkom/ Crab [केंकड़ा/काकेड़ा]	
G.15	Sursuri [सुरसुरी]		**I**	**Fish (Indigenous)**	
G.16	Ghaghar [घाघर]		I.1	Pothi [पोठी]	
G.17	Maina [मैना]		I.2	Ichcha/Chingri [इच्चा/चिंगरी]	
G.18	Putam [पुटम]		I.3	Koronjo [कोरोंजो]	
G.19	Askal [असकल]		I.4	Bale [बाले]	
G.20	Burdliyad/Burdulu/Ufia [बर्द्लियद]		I.5	Budu [बुडू]	
G.21	Dhamna [धामना]		I.6	Noya/Kucheela [नोया/कुचीला]	
G.22	Batakh/Gede [बत्तख/गेडे]		I.7	Setua/Keyosuti [सेतुआ/केयोसुटी]	
G.23	Haau(Lalchiti) [हाऊ/लालचिटी]		I.8	Linda [लिंडा]	
G.24	Squirrel [गिलहरी]		I.9	Getumachli [गेतुमछली]	

1=Every day (>2 times) 2=Every day (2 times) 3=Every day (1 time) 4=5 to 6 days/week 5=3 to 4 days /week 6=1 to 2 days/ week 7=Once in a fortnight 8=Once in a month 0=Never

S. No.	Food Items	Frequency of Intake	S. No.	Food Item	Frequency of Intake
I.10	Hadad hai/ Chirpi machli [हदाद हाई/ चिरपी मछली]		J.6	Coconut [नारीयल]	
I.11	Chudhako/Chodha [चुधाको/चोधा]		J.7	Mango [आम]	
I.12	Aira [आईरा]		J.8	Mulberry/Toot [शहतूत/तूत]	
I.13	Kakandahayi [ककंडाहाई]		J.9	Sinju /Bael fruit [बेल फल]	
I.14	Binghayi [बींगहायी]		J.10	Grapes	
I.15	Madsakam [मदसकम]		**K**	**Fruits (Indigenous)**	
I.16	Sundi [सुंडी]		K.1	Tarop/Char [तारोप/चार]	
I.17	Loa suti (snail) [लोया सूती]		K.2	Kusum/Baru [कुसुम]	
I.18	Genger [गेंगेर]		K.3	Amda/Amru [अमडा/अमरु]	
I.19	Dry fish		K.4	Tiril/Kendu [तीरिल/केन्दु]	
**J**	**Fruits (Non-Indigenous)**		K.5	Dhela [ढेला]	
J.1	Guava /Tamras [अमरुद/तामरस]		K.6	Podo /Fig [पोडो]	
J.2	Amrit/Papaya [अमृत/ पपीता]		K.7	Loa/Dumur [लोवा]	
J.3	Apple [सेब]		**L**	**Green Leafy Vegetables (Non-Indigenous)**	
J.4	Banana [केला]		L.1	Mint [पुदीना]	
J.5	Amla [आमला]		L.2	Coriander [धनिया]	

1=Every day (>2 times) 2=Every day (2 times) 3=Every day (1 time) 4=5 to 6 days/week 5=3 to 4 days /week 6=1 to 2 days/ week 7=Once in a fortnight 8=Once in a month 0=Never

S. No.	Food Items	Frequency of Intake	S. No.	Food Item	Frequency of Intake
L.3	Muri arak/ Radish greens [मूली साग]		M.14	Lupu arak [लुपूआड़ा]	
L.4	Palak		M.15	Lal bhaji [लाल भाजी]	
**M**	**Green Leafy Vegetables (Indigenous)**		M.16	Urile arak [उरिलेआड़ा]	
M.1	Saru arak (kaanda araha) [सारुआड़ा /कांदाड़ा]		M.17	Lundi arak [लुंडीआड़ा]	
M.2	Munga arak/sajna [मूंगाड़ा]		M.18	Soredhe/ Bir/Rimil [सोरेधे/बीर/रीमिल आड़ा]	
M.3	Kantha arak [कंथाड़ा]		M.19	Jojo arak [जोजो आड़ा]	
M.4	Dail ara [डाईलड़ा]		M.20	Charmani arak [चर्मणिआड़ा]	
M.5	Saangadha/Kaanda saag[सान्गधा/कांदा साग]		M.21	Kauwa arak [कउवाआड़ा]	
M.6	Sirgiti arak [सिरगिट्टीआड़ा]		M.22	Kotle arak [कोटलेआड़ा]	
M.7	Aloo arak [आलूआड़ा]		M.23	Sugu arak [सुगुआड़ा]	
M.8	Garundi arak [गरोंदीआड़ा]		M.24	Uli arak [उलीआड़ा]	
M.9	Upundu arak [उपुंडूआड़ा]		M.25	Tir arak [तीरआड़ा]	
M.10	Ohio arak [ओहियोआड़ा]		M.26	Lasodar /Lasodh arak [लसोदर/लसोधाड़ा]	
M.11	Hesa arak [हेसाड़ा]		M.27	Biyur arak/Netho saag [बियुरआड़ा / नेथो साग]	
M.12	Susni arak[सुसनीआड़ा]		M.28	Chaina palak [चाइना पालक]	
M.13	Budilae ara [बुडीलाईआड़ा]		M.29	Phutkal saag	

1=Every day (>2 times) 2=Every day (2 times) 3=Every day (1 time) 4=5 to 6 days/week 5=3 to 4 days /week 6=1 to 2 days/ week 7=Once in a fortnight 8=Once in a month 0=Never

S. No.	Food Items	Frequency of Intake	S. No.	Food Item	Frequency of Intake
M.30	Leped arak		N.12	Lady's Finger [भिंडी]	
M.31	Beng saag		N.13	Jackfruit [कटहल]	
M.32	Sing ara/ Konra saag		N.14	Cabbage [पत्ता गोभी]	
M.33	Kalai saag		N.15	Kundri [कुंदरी]	
**N**	**Vegetables (Non-Indigenous)**		N.16	Faliya Parasbin/ french beans	
N.1	Kaera/ Green plantain [कैरा]		N.17	Parwal	
N.2	Kundri /kundru [कुंदरू]		**O**	**Vegetables (Indigenous)**	
N.3	Papaya/Man kunda [पपीता/ मन कुंडा]		O.1	Field beans/Malhan/Sem/Simbi [मलहान/सेम/सिम्बी]	
N.4	Drumstick [सैजन की फली]		O.2	Rugra [रुगरा]	
N.5	Tomato [टमाटर]		O.3	Dodo [डोडो]	
N.6	Bitter Gourd/ Karela [करेला]		O.4	Ketha [केथा]	
N.7	Bottle Gourd [लौकी]		O.5	Bir karela [बीर करेला]	
N.8	Bengar /Brinjal [बैंगन]		O.6	Hutarba [हुटारबा]	
N.9	Ridge gourd/ Jhinga [तुरई/ झिंगला]		O.7	Singra [सिंगड़ा]	
N.10	Pumpkin/Kumda/Kaddu/Kohda [कद्दू]		O.8	Kundri	
N.11	Cucumber[खीरा]		**P**	**Mushrooms (Indigenous)**	

1=Every day (>2 times) 2=Every day (2 times) 3=Every day (1 time) 4=5 to 6 days/week 5=3 to 4 days /week 6=1 to 2 days/ week 7=Once in a fortnight 8=Once in a month 0=Never

S. No.	Food Items	Frequency of Intake	S. No.	Food Items	Frequency of Intake
P.1	Kurthi ud [कुरथी उद्]		P.17	Pataka ud [पटका उद्]	
P.2	Koyaansakam [कोयांसकाम]		P.18	Kunda ud/Koode ud [कुंदा उद्]	
P.3	Putkal [पुटकल]		P.19	Tormoda ud [तोर्मोदा उद्]	
P.4	Simdali ud [सिमदडली उद्]		P.20	Gomda ud [गोमदा उद्]	
P.5	Gitli ud [गितली उद्]		P.21	Badai ud [बड़ाय उद्]	
P.6	Koode ud [कूदे उद्]		P.22	Simdali ud [सिमदाली उद्]	
P.7	Piri ud [पिधि उद्]		P.23	Rugra/Putkui [रूगरा/पुटकुई]	
P.8	Bunum ud [बुनुम उद्]		P.24	Jati Putkui (Black) [जाति पुटकुई]	
P.9	Indi ud [इंदी उद्]		P.25	Pundi Putkui (White) [पुंडी पुटकुई]	
P.10	Gende ud [गेंडे]		P.26	Aata ud [आटा उद्]	
P.11	Tumba ud [तुम्बा उद्]		P.27	Rampatka ud [रामपटका उद्]	
P.12	Chokerotte [चोकेरोटे]		P.28	Burunda	
P.13	Lechde ud [लेचेड़े उद्]		**Q**	**Roots and Tubers (Non-Indigenous)**	
P.14	Dasayi ud [दसायी उद्]		Q.1	Garlic [लहसुन]	
P.15	Badhai ud/Badhe ud [बढाय उद्]		Q.2	Onion [प्याज़]	
P.16	Beng putu [बेंग पुटू]		Q.3	Potato [आलू]	

1=Every day (>2 times) 2=Every day (2 times) 3=Every day (1 time) 4=5 to 6 days/week 5=3 to 4 days /week 6=1 to 2 days/ week 7=Once in a fortnight 8=Once in a month 0=Never

S. No.	Food Items	Frequency of Intake	S. No.	Food Items	Frequency of Intake
Q.4	Radish [मूली/मुराइ]		S.5	Mustard oil [सरसों का तेल]	
**R**	**Roots and tubers (Indigenous)**		S.6	Soyabean oil	
R.1	Ol/pinde **[**ओल**]**		**T**	**Oils and Fat (Indigenous)**	
R.2	Haraad bo [हराद बो]		T.1	Turi/Desi mustard oil [सरसों का तेल]	
R.3	Haseaar sanga [हसीआर संगा]		T.2	Coconut oil [नारियल का तेल]	
R.4	Adel sanga [एदेल संगा]		T.3	Redi Oil/Arhandi Oil [अरहंडी का तेल]	
R.5	Saaru [सारू]		T.4	Kuindi Oil/Mahua [महुआ का तेल]	
R.6	Toti [टोटी]		T.5	Kusum oil [कुसुम का तेल]	
R.7	Kondi sanga [कोंडी संगा]		T.6	Neem oil [नीम का तेल]	
R.8	Koolarumpa [कूलारुम्पा]		T.7	Karanj oil [करंज का तेल]	
R.9	Maisarsanga [मैसरसंगा]		T.8	Kujri oil [कुजरी का तेल]	
**S**	**Oils and Fat (Non-Indigenous)**		T.9	Surgunja oil [सुरगुंजा का तेल]	
S.1	Refined vegetable oil [रिफाइंड तेल]		**U**	**Nuts and oilseeds**	
S.2	Ghee (Cow/Buffalo) [घी- गाएं/भैंस]		U.1	Groundnuts [मूंगफली]	
S.3	Vanaspati/ Dalda [वनस्पति/डालडा]		U.2	Coconuts [नारियल]	
S.4	Groundnut oil [मूंगफली का तेल]		U.3	Almond	

1=Every day (>2 times) 2=Every day (2 times) 3=Every day (1 time) 4=5 to 6 days/week 5=3 to 4 days /week 6=1 to 2 days/ week 7=Once in a fortnight 8=Once in a month 0=Never

S. No.	Food Items	Frequency of Intake	S. No.	Food Items	Frequency of Intake
**V**	**Sugar**		X.3	Tea [चाय]	
V.1	Sugar(शक्कर)		X.4	Sweets (Balushai/Ladoo/Malpua) [मिठाई-बलुशाई/लड्डू/मालपुआ]	
V.2	Brown sugar [लाल शक्कर]		X.5	Muri ghugni [मुड़ी-घुगनी]	
V.3	Jaggery [गुड़]		X.6	Kheer [खीर]/ Barfi [बर्फी]	
V.4	Honey [शहद]		X.7	Jalebi [जलेबी]	
**W**	**Alcohol**		X.8	Pav roti [पाव रोटी]	
W.1	Handiya [हांडिया]		X.9	Bhujia/Mixture/ Papad/Sev [भुजिया/ सेव/मिक्सचर/पापड़]	
W.2	Khijur Taadi [खजूर ताड़ी]		X.10	Aloo Chop/ Samosa/ Dhuska/ Idli [आलू चॉप/ सामोसा/ धुस्का/ इडली]	
W.3	Mahua Taadi [महुआ ताड़ी]		X.11	Chips/Kachori/Mathri/Namkeen/Nimki [चिप्स /कचोरी/मठरी/नमकीन/निमकी]	
W.4	Oraiya Bakla [औरैया बकला]		X.12	Aloo Pakora/ Bread Pakora [आलू पकोड़ा/ ब्रेड पकोड़ा]	
W.5	Tukui Handi [तुकुई हांड़ी]		X.13	Biscuit, sweet [बिस्कुट, मीठा]	
W.6	English [अंग्रेज़ी]		X.14	Biscuit, salty [बिस्कुट, नमकीन]	
**X**	**Miscellaneous (Non-Indigenous)**		X.15	Chowmein [चाऊमीन]	
X.1	Mixed Pickle		X.16	Chocolate [चाकलेट]	
X.2	Mango pickle				

1=Every day (>2 times) 2=Every day (2 times) 3=Every day (1 time) 4=5 to 6 days/week 5=3 to 4 days /week 6=1 to 2 days/ week 7=Once in a fortnight 8=Once in a month 0=Never

2b. Munda Food Frequency Questionnaire (Last 1 month) -Winter Season

Household ID: | | | | | | | | | | |

Date: | | | | | | |

Season ___________________________

S. No.	Food Items	Frequency of Intake	S. No.	Food Item	Frequency of Intake
**A**	**Cereals (Non-Indigenous)**		B.8	Raasdhan [रासधान]	
A.1	Rice, Hybrid [चावल,हाईब्रिड]		B.9	Minjri [मिन्जरी]	
A.2	Guhum/ Wheat flour [गेंहू/गुहम]		B.10	Aara/Aarodhan [अरोधान]	
A.3	Bajra/Gangayi [बाजरा/गंगाई]		B.11	Jolpo baba [जोल्पोबाबा]	
A.4	Muri/ Puffed Rice [मूड़ी]		B.12	Gilti baba [गिलटीबाबा]	
A.5	Corn/Makkai,Hybrid [मक्का/ मक्कई, हाईब्रिड]		B.13	Pundi goda [पुंडी गोडा]	
A.6	Rice Flakes/ chirwa [चिडवा]		B.14	Rieci baba [राइसी बाबा]	
A.7	Pioneer dhan		B.15	Karnidhan [कर्निधान]	
A.8	Diku baba		B.16	Lalatdhan [ललाटधान]	
**B**	**Cereals (Indigenous)**		B.17	Panchanwayedhan [पचानवेधान]	
B.1	Laaldhan/Arababa [लालधान/आराबाबा]		B.18	Dusridhan [दुसरिधान]	
B.2	Safeddhan [सफेदधान]		B.19	Hathipanjardhan [हाथीपंजरधान]	
B.3	Jedengdhan [जेदेंगधान]		B.20	Rajdhan [राजधान]	
B.4	Daanidhan [दानीधान]		B.21	Sambalpuri [संबलपुरी]	
B.5	Pasoda baba [पसडा बाबा]		B.22	Garmi dhan/teba baba [गरमाधान/ टेवाँ बाबा]	
B.6	Karangadhan [करांगाधान]		B.23	Pagla Goda [पगला गोड़ा]	
B.7	Kannaudhan [कन्नौधान]		B.24	Sorno baba [सोरनो बाबा]	

1=Every day (>2 times) 2=Every day (2 times) 3=Every day (1 time) 4=5 to 6 days/week 5=3 to 4 days /week 6=1 to 2 days/ week 7=Once in a fortnight 8=Once in a month 0=Never

S. No.	Food Item	Frequency of Intake	S. No.	Food Item	Frequency of Intake
B.25	Gudlu [गुड़लु]		D.1	Suthro [सुथरी]	
B.26	Anthanawaye dhan [अनठानव्वे धान]		D.2	Khesari dal [केसरी दाल]	
B.27	Bajra/Gangayi [बाजरा/गंगई]		D.3	Baturi/Tiri riti [बाटूरी/तीरि रीती]	
B.28	Mandua/Kodhe/Janhe [मंडुआ/कोड़ेह/जन्हे]		D.4	Danbudi/Ghangra [डंगबुडी/घांघरा]	
B.29	Mahua dhan		D.5	Raher/Red gram (whole) [अरहर/रहरी/रहेड छिलके वाली]	
B.30	Naniha		D.6	Raher/ Red gram (dehusked) [रहेड, बिना छिलके वाली]	
B.31	Mota dhan		D.7	Boot/ Bengal gram dal [बूट/चना]	
B.32	Maniya dhan		D.8	Kulthi [कुल्थी]	
B.33	Munadhan		D.9	Rambada dal/Urad/Black gram (whole) [रामबडा छिलके वाली]	
B.34	Maize		D.10	Rambada dal/Urad/Black gram (dehusked) [रामबडा बिना छिलके वाली]	
**C**	**Pulses (Non-Indigenous)**		**E**	**Milk and Milk products**	
C.1	Raher/Red gram (whole) [अरहर/रहरी/रहेड,छिलके वाली]		E.1	Cow's Milk [गाय का दूध]	
C.2	Raher/ Red gram (dehusked) [अरहर/रहरी/रहेड,बिना छिलके वाली]		E.2	Buffalo's Milk [भेंस का दूध]	
C.3	Masoor dal [मसूर]		E.3	Powder milk	
C.4	Boot/ Bengal gram dal [बूट/चना]		E.4	Packet milk	
C.5	Moong/green gram (whole) [मूंग, छिलके वाली]		**F**	**Meat and Meat Products (Non-Indigenous)**	
C.6	Moong/green gram (dehusked) [मूंग, बिना छिलके वाली]		F.1	Meat/Mutton [मीट/बकरी/भेड़ का मांस]	
C.7	Rambada dal/ Black gram/Urad (whole) [रामबडा छिलके वाली]		F.2	Beef [गाय का मांस]	
C.8	Rambada dal/Black gram/Urad (dehusked) [रामबडा बिना छिलके वाली]		F.3	Chicken [मुर्गा]	
**D**	**Pulses (Indigenous)**		F.4	Eggs [अंडा]	

1=Every day (>2 times) 2=Every day (2 times) 3=Every day (1 time) 4=5 to 6 days/week 5=3 to 4 days /week 6=1 to 2 days/ week 7=Once in a fortnight 8=Once in a month 0=Never

S. No.	Food Item	Frequency of Intake	S. No.	Food Item	Frequency of Intake
F.5	Pig [सूअर]		G.20	Burdliyad/Burdulu/Ufia [बुर्द्लियद/बुर्दुलू/उफिया]	
F.6	Rabbit [खरगोश/कुलय]		G.21	Dhamna [धामना]	
F.7.	Squirrel [गिलहरी]		G.22	Batakh/Gede [बत्तख/गेडे]	
**G**	**Meat and Meat Products (Indigenous)**		G.23	Haau(Lalchiti) [हाऊ/लालचिटी]	
G.1	Jangli murgi [जंगली मुर्गी]		G.24	Gudu	
G.2	Jungli Peacock [जंगली मोर]		**H**	**Fish (Non-Indigenous)**	
G.3	Tumbuli (insect) [तुमबुली]		H.1	Katla [कतला]	
G.4	Neelirasi [नीली रासी]		H.2	Rohu [रोहू/रूही]	
G.5	Jangli suar [बीर सुकरी/जंगली सूअर/सिलीब]		H.3	Iccha/Chingri [इच्चा/चिंगरी]	
G.6	Porcupine / Saahi/Jikki/Torod [किस्सा/साही/जिक्की/तोरोद]		**I**	**Fish (Indigenous)**	
G.7	Bando [बंडो]		I.1	Pothi [पोठी]	
G.8	Hadhoga/Siyar [हाधोगा/सीयार]		I.2	Koronjo [कोरोंजो]	
G.9	Pigeon /Kabutar [कबूतर]		I.3	Bale [बाले]	
G.10	Bees/ Madumakkhi [मधुमक्खी/मधुरस]		I.4	Budu [बुडू]	
G.11	Chitri [चित्री]		I.5	Setua/Keyosuti [सेतुआ/केयोसुटी]	
G.12	Duhur [दुहुर]		I.6	Linda [लिंडा]	
G.13	Sursuri [सुरसुरी]		I.7	Sundi/Getumachli [सुंडी/ गेतुमछली]	
G.14	Ghaghar [घाघर]		I.8	Hadad hai/ Chirpi machli [हदाद हाई/चिरपी मछली]	
G.15	Maina [मैना]		I.9	Chudhako/Chodha [चुधाको/चोधा]	
G.16	Putam [पुटम]		I.10	Aira [आईरा]	
G.17	Khasi [खासी]		I.11	Kakandahayi [ककंडाहाई]	
G.18	Demta [डेमटा]		I.12	Binghayi [बींगहायी]	
G.19	Askal [असकल]		I.13	Madsakam [मदसकम]	

1=Every day (>2 times) 2=Every day (2 times) 3=Every day (1 time) 4=5 to 6 days/week 5=3 to 4 days /week 6=1 to 2 days/ week 7=Once in a fortnight 8=Once in a month 0=Never

S. No.	Food Items	Frequency of Intake	S. No.	Food Item	Frequency of Intake
I.14	Genger [गेंगेर]		L.4	Mustard leaves/Maani arak [सरसों साँग/मणिआड़ा]	
I.15	Dry fish		L.5	Onion leaves [प्याज़ के पत्ते]	
I.16	Dry pot		L.6	Bathua leaves [बथुआ के पत्ते]	
**J**	**Fruits (Non-Indigenous)**		L.7	China palak	
J.1	Guava /Tamras [अमरुद/तामरस]		L.8	Palak [पालक]	
J.2	Amrit/Papaya [अमृत/पपीता]		**M**	**Green Leafy Vegetables (Indigenous)**	
J.3	Apple [सेब]		M.1	Saru arak/ kaanda arak [सारुआड़ा/कांदाड़ा]	
J.4	Banana [केला]		M.2	Munga arak/sajna [मूंगाड़ा]	
J.5	Amla [आमला]		M.3	Aloo arak [आलूआड़ा]	
J.6	Coconut [नारीयल]		M.4	Lehsun saag [लहसुन साँग/ रेह्सुनीआड़ा]	
J.7	Custard Apple [शरीफा]		M.5	Chaari arak [चारीआड़ा]	
J.8	Grapes [अंगूर]		M.6	Chiringid arak [चिरिंगिदआड़ा]	
J.9	Orange [संतरा]		M.7	Chimti/ Mui arak [चिमटी/ मुईआड़ा]	
J.10	Pomegrenate [अनार]		M.8	Sarla /Serali arak [सरला साँग/ सेरालीआड़ा]	
**K**	**Fruits (Indigenous)**		M.9	Mattha saag [मट्ठा साँग]	
K.1	Amda/Amru [अमडा/अमरु]		M.10	Chana saag/Bengal gram leaves [बूटआड़ा]	
K.2	Ber [बेर/डोडाडी]		M.11	Amaranth/Leper arak [लेपरआड़ा]	
K.3	Miril/Mirle [मीरिल/मीरले]		M.12	Khesari saag [खेसारी साँग]	
K.4	Kudrum/Jojo Fruit [जोजो फल]		M.13	Posta arak [पोस्ताआड़ा]	
K.5	Loa [लोया]		M.14	Teeri-riti arak [तीरी-रीतीआड़ा]	
K.6	Banyan fruit [बरगद का फल]		M.15	Losodar arak [लोसोदारआड़ा]	
**L**	**Green Leafy Vegetables (Non-Indigenous)**		M.16	Heteteyo arak [हेतेतेयोआड़ा]	
L.1	Mint [पुदीना]		M.17	Biyur arak [बीयुरआड़ा]	
L.2	Coriander [धनिया]		M.18	Chatom arak [छातोमआड़ा]	
L.3	Muri arak/ Radish greens [मूली साँग/ मूरीआड़ा]		M.19	Chakod/choke ara [चकोड/चोक्केआड़ा]	

1=Every day (>2 times) 2=Every day (2 times) 3=Every day (1 time) 4=5 to 6 days/week 5=3 to 4 days /week 6=1 to 2 days/ week 7=Once in a fortnight 8=Once in a month 0=Never

S. No.	Food Item	Frequency of Intake	S. No.	Food Items	Frequency of Intake
M.20	Kudrum saag/jojo arak[कुद्रुम साँग/ जोजोआड़ा]		O.7	Burju baha [बुर्जू बाहा]	
M.21	Sanai saag		O.8	Sanai /Jindiba phool [सनाइ/जिंदिबा फूल]	
M.22	Sweet potato leaves [शकरकंद के पत्ते]		O.9	Barbatti vegetable/Bodi [बरबट्टी/बोडी]	
M.23	Pumpkin leaves [कद्दू के पत्ते]		O.10	Kudrum flower	
M.24	Muchdi ara		**P**	**Roots and Tubers (Non-Indigenous)**	
**N**	**Vegetables (Non-Indigenous)**		P.1	Garlic [लहसुन]	
N.1	Kaera/ Green plantain [कैरा]		P.2	Onion [प्याज़]	
N.2	Papaya/Man kunda [पपीता/ मन कुंडा]		P.3	Potato [आलू]	
N.3	Cauliflower [फुलगोबी]		P.4	Radish [मूली/मुराइ]	
N.4	Tomato [टमाटर]		P.5	Carrot [गाजर]	
N.5	Bitter Gourd/ Karela[करेला]		**Q**	**Roots and tubers (Indigenous)**	
N.6	Bengar /Brinjal [बैंगन]		Q.1	Adel sanga [अदेल संगा]	
N.7	Pumpkin/Kumda/Kaddu/Kohda [कद्दू]		Q.2	Haseaar sanga [हसीआर संगा]	
N.8	Cucumber[खीरा]		Q.3	Jat sanga [जाट संगा]	
N.9	Lady's Finger [भिंडी]		Q.4	Kondi sanga [कोंडी संगा]	
N.10	Cabbage [पत्ता गोभी]		Q.5	Maisarsanga [मैसरसंगा]	
N.11	Bottle gourd		Q.6	Koolarumpa [कूलारुम्पा]	
**O**	**Vegetables (Indigenous)**		Q.7	Ole [ओल]	
O.1	Bir karela [बीर करेला]		Q.8	Marom sanga	
O.2	Kundri /Kundru [कुंदरू]		Q.9	Saru sanga	
O.3	Ridge gourd/ Jhinga [तुरई/ झिंगला]		**R**	**Oils and Fat (Non-Indigenous)**	
O.4	Dodo [डोडो]		R.1	Refined vegetable oil [रिफाइंड तेल]	
O.5	Field beans/Malhan/Sem/Simbi [मलहान/सेम/सिम्बी]		R.2	Ghee (Cow/Buffalo) [घी- गाएं/भेंस]	
O.6	Hutarba [हुटारबा]		R.3	Vanaspati/ Dalda [वनस्पति/डालडा]	

1=Every day (>2 times) 2=Every day (2 times) 3=Every day (1 time) 4=5 to 6 days/week 5=3 to 4 days /week
6=1 to 2 days/ week 7=Once in a fortnight 8=Once in a month 0=Never

S. No.	Food Item	Frequency of Intake	S. No.	Food Item	Frequency of Intake
R.4	Groundnut oil [मूंगफली का तेल]		W.3	Sweets (Balushai/Ladoo/Malpua/Gulgula) [मिठाई-बलुशाई/लड्डू/मालपुआग/गुलगूला]	
R.5	Mustard oil [सरसों का तेल]		W.4	Muri ghugni [मुड़ी-घुगनी]	
**S**	**Oils and Fat (Indigenous)**		W.5	Kheer [खीर]/ Barfi [बर्फी]	
S.1	Turi/Desi mustard oil[सरसों का तेल]		W.6	Jalebi [जलेबी]	
S.2	Coconut oil [नारियल का तेल]		W.7	Pav roti [पाव रोटी]	
S.3	Kusum oil [कुसुम का तेल]		W.8	Bhujia/Mixture/ Papad/Sev [भुजिया/ सेव/मिक्सचर/पापड़]	
S.4	Surgunja oil [सुरगुंजा का तेल]		W.9	Aloo Chop/ Samosa/ Dhuska/ Idli [आलू चॉप/ सामोसा/ धुस्का/ इडली]	
**T**	**Nuts and oilseeds**		W.10	Chips/Kachori/Mathri/Namkeen/Nimki [चिप्स /कचोरी/मठरी/नमकीन/निमकी]	
T.1	Groundnuts [मूंगफली/बदाम]		W.11	Aloo Pakora/ Bread Pakora [आलू पकोड़ा/ ब्रेड पकोड़ा]	
T.2	Coconuts [नारियल]		W.12	Biscuit, sweet [बिस्कुट, मीठा]	
**U**	**Sugar**		W.13	Biscuit, salty [बिस्कुट, नमकीन]	
U.1	Sugar(शक्कर)		W.14	Chowmein [चाऊमीन]	
U.2	Brown sugar [लाल शक्कर]		W.15	Chocolate [चाकलेट]	
U.3	Jaggery [गुड़]				
U.4	Honey [शहद]				
**V**	**Alcohol**				
V.1	Handiya [हांडिया]				
V.2	Khijur Taadi [खजूर ताड़ी]				
V.3	Mahua Taadi [महुआ ताड़ी]				
V.4	Tukui Handi [तुकुई हांड़ी]				
V.5	English [अंग्रेज़ी]				
**W**	**Miscellaneous (Non-Indigenous)**				
W.1	Pickle (…………………………) [अचार]				
W.2	Tea [चाय]				

1=Every day (>2 times) 2=Every day (2 times) 3=Every day (1 time) 4=5 to 6 days/week 5=3 to 4 days /week 6=1 to 2 days/ week 7=Once in a fortnight 8=Once in a month 0=Never
