# Supplementary material for: Indigenous food environment and dietary patterns of Munda community of Jharkhand, India
Source: BMC Nutr. 2025 Oct 21;11:189. doi: 10.1186/s40795-025-01159-2 (PMC12539013; doi:10.1186/s40795-025-01159-2)
Supplement: Supplementary file 3 — Supplementary Material 3 [file 40795_2025_1159_MOESM3_ESM.docx]

Supplementary File 3a

64 food sub-categories for monsoon season

| **S.No.** | **Categories** | **Food Item Code** |
| --- | --- | --- |
| 1. | Commonly consumed non-indigenous cereal staples | Rice, Hybrid  Guhum/Wheat  Whole wheat flour |
| 2. | Less-commonly consumed non-indigenous cereals | Corn/Makkai, Hybrid |
| 3. | Non-indigenous Millets | Bajra/Gangayi |
| 4. | Non-indigenous Cereals eaten as a snack | Muri/Puffed Rice  Rice Flakes/chirwa |
| 5. | Commonly consumed indigenous cereal staples | Laaldhan/Arababa  Safeddhan  Jedengdhan  Karanga dhan  Gilti baba  Pundi goda  Rieci baba  Karnidhan  Lalatdhan  Panchanwaye dhan  Dusridhan  Sambalpuri  Garmi dhan/teba baba  Pagla Goda  Sorno baba  Gudlu  Mansuri |
| 6. | Less-commonly consumed indigenous cereals | Daanidhan  Pasoda baba  Kannaudhan  Raasdhan  Minjri  Aara/Aarodhan  Jolpo baba  Hathipanjardhan  Rajdhan  Anthanawaye dhan  Sankar dhaan |
| 7. | Indigenous Millets | Bajra/Gangayi  Mandua/Kodhe/Janhe |
| 8. | Commonly consumed non-indigenous pulses | Raher/Red gram (whole)  Raher/Red gram (dehusked)  Masoor dal  Moong/green gram (whole) |
| 9. | Less commonly consumed non-indigenous pulses | Kulthi  Boot/Bengal gram dal  Moong/green gram (dehusked)  Rambada dal/ Black gram/Urad (whole)  Rambada dal/Black gram/Urad (dehusked)  Rajma |
| 10. | Commonly consumed indigenous pulses | Moong/green gram (Dehusked)  Kulthi  Rambada dal/Urad/Black gram (dehusked) |
| 11. | Less commonly consumed indigenous pulses | Suthro  Kesari dal  Baturi/Tiri riti  Danbudi/Ghangra  Raher/Red gram (whole)  Raher/Red gram (dehusked)  Boot/Bengal gram dal  Masoor dal  Moong/green gram (whole)  Rambada dal/Urad/Black gram (Whole)  Bodi |
| 12. | Milk and milk products from the market | Cow’s milk  Buffalo’s milk  Packaged Milk  Paneer  Curd |
| 13. | Non-indigenous meat products from reared livestock | Chicken  Eggs |
| 14. | Non-indigenous meat products from market | Meat/Mutton  Beef  Pig  Khasi |
| 15. | Indigenous meat products from forest/field | Jangli murgi  Rabbit  Jungli Peacock  Tumbuli (insect)  Neelirasi  Field rat/Musa/Guddu  Jangli suar  Porcupine/Saahi/Jikki/Torod  Bando  Hadhoga (Siyar)  Pigeon (Kabutar)  Bees/Madumakkhi  Chitri  Duhur  Sursuri  Ghaghar  Maina  Putam  Askal  Burdliyad/Burdulu/Ufia  Dhamna  Haau (Lalchiti)  Squirrel  Demta |
| 16. | Indigenous meat products from reared livestock | Batakh/Gede |
| 17. | Non-indigenous fish | Katla  Rohu |
| 18. | Non-indigenous molluscs | Katkom/Crab |
| 19. | Commonly consumed indigenous fish from water bodies | Pothi  Ichcha Chingri  Koronjo  Bale  Budu  Getumachli  Hadad hai/Chirpi machli  Chudhako/Chodha  Sundi  Dry fish |
| 20. | Less commonly consumed indigenous fish from water bodies | Noya/Kucheela  Linda  Aira  Kakandahayi  Binghayi  Madsakam  Genger |
| 21. | Commonly consumed indigenous molluscs from water bodies | Setua/Keyosuti  Loa suti (snail) |
| 22. | Non-indigenous fruits from roadside and wasteland | Guava/Tamras  Amrit/Papaya  Sinju/Bael fruit  Mango  Mulberry/Toot |
| 23. | Non-indigenous fruits from market | Apple  Banana  Amla  Coconut  Grapes |
| 24. | Indigenous fruits from forests and/or open spaces | Tarop/Char  Kusum/Baru  Amda/Amru  Tiril/Kendu  Dhela  Podo/Fig  Loa/Dumur |
| 25. | Non-indigenous GLV commonly consumed from market | Palak  Muri arak/ Radish greens  Chaina palak |
| 26. | Non-indigenous GLV consumed as condiments/herbs | Mint  Coriander |
| 27. | Commonly consumed Indigenous GLV obtained from forest | Saru arak (kaanda araha)  Sirgiti arak  Jojo arak  Kauwa arak  Phutkal saag  Sing ara/Konra saag  Singra |
| 28. | Less commonly consumed Indigenous GLV obtained from forest | Ohio arak  Hesa arak  Soredhe/Bir/Rimil  Lasodar/Lasodh arak  Biyur arak/Netho saag |
| 29. | Commonly consumed Indigenous GLV growing as weeds | Susni arak  Kalai saag |

| 30. | Less-commonly consumed indigenous GLV growing as weeds | Kantha arak  Upundu arak  Lupu arak  Tir arak |
| --- | --- | --- |
| 31. | Commonly consumed Indigenous GLV cultivated | Munga arak/Sajna  Saangadha/Kaanda saag  Garundi arak  Charmani arak  Kotle arak  Leped arak  Beng saag |
| 32. | Less commonly consumed Indigenous GLV cultivated | Dail ara  Aloo arak  Budilae ara  Lundi arak  Lal bhaji  Urile arak  Sugu arak  Uli arak |
| 33. | Non-indigenous vegetables from market | Tomato  Bittergourd/karela  Bottle gourd  Bengar/Brinjal  Ridge gourd/Jhinga  Cucumber  Lady’s finger  Cabbage  Faliya Parasbin/french beans  Parwal |
| **S.No.** | **Categories** | **Food Item Code** |
| 34. | Non-indigenous vegetables cultivated | Papaya/Man kunda  Pumpkin/Kumda/Kaddu/Kohda  Kundri/Kundru  Kundri |
| 35. | Non-indigenous vegetables from open spaces | Jackfruit  Drumstick  Kaera/Green plantain |
| 36. | Less commonly consumed indigenous vegetables from forests | Bir karela  Hutarba |
| 37. | Commonly consumed Indigenous vegetables from market | Field beans/Malhan/Sem/Simbi  Dodo |
| 38. | Commonly consumed Indigenous vegetables cultivated | Kundri |
| 39. | Less commonly consumed indigenous vegetables cultivated | Ketha |
| 40. | Commonly consumed mushrooms | Rugra  Koyaansakam  Simdali ud  Gitli ud  Koode ud  Piri ud  Indi ud  Tumba ud  Lechde ud  Badhai ud/Badhe ud  Pataka ud  Kunda ud/Koode ud  Tormoda ud  Badai ud  Simdali ud  Rugra/Putkui  Jati Putkui (Black)  Pundi Putkui (White)  Rampatka ud  Burunda |
| 41. | Less-commonly consumed mushrooms | Kurthi ud  Bunum ud  Gende ud  Chokerotte  Dasayi ud  Beng putu  Gomda ud  Aata ud |
| 42. | Non-indigenous roots and tubers as a vegetable preparation from market | Potato  Radish |
| 43. | Non-indigenous roots and tubers used as a condiment from market | Garlic  Onion |
| 44. | Non-indigenous roots and tubers cultivated | Kondi sanga |
| 45. | Commonly consumed indigenous tubers from forest | Haraad bo  Toti  Saaru |
| 46. | Less commonly consumed indigenous tubers from forest | Haseaar sanga  Adel sanga  Koolarumpa  Maisarsanga |
| 47. | Commonly consumed indigenous tubers cultivated | Ol/pinde |
| 48. | Non-indigenous saturated oils and fats from market | Ghee (Cow/Buffalo)  Vanaspati/Dalda |
| 49. | Non-indigenous unsaturated oils from market | Refined vegetable oil  Soyabean oil |
| 50. | Non-indigenous unsaturated oils derived from pressing seeds | Groundnut Oil  Mustard Oil |
| 51. | Home produced indigenous oils and fats | Turi/Desi mustard oil  Coconut oil  Redi Oil/Arhandi Oil  Kuindi Oil/Mahua  Kusum Oil  Neem Oil  Karanj Oil  Kujri oil  Surguja oil |
| 52. | Nuts and oilseeds from Govt. programme | Groundnuts |
| 53. | Nuts and oilseeds from market | Coconuts  Almonds |
| 54. | Sugars from forest | Honey |
| 55. | Sugars from market | Brown sugar  Jaggery |
| 56. | Sugars from TPDS | Sugar |
| 57. | Alcohols prepared locally | Handiya  Khijur taadi  Mahua taadi  Oraiya Bakla  Tukui Handi |
| 58. | Alcohols bought from the market | English |
| 59. | Miscellaneous foods eaten as a side dish | Mixed pickle  Mango pickle |
| 60. | Cooked Sweets and desserts | Kheer |
| 61. | Freshly prepared dishes from the market | Muri ghungni  Aloo Chop/Samosa/Dhuska/Idli  Aloo Pakora/Bread Pakora  Chowmein |
| 62. | Packaged foods | Pav roti  Bhujia/Mixture/Papad/Sev  Chips/Kachori/Mathri/Nmakeen/Nimki  Biscuit, sweet  Biscuit, salty  Chocolate |
| 63. | Hot beverage | Tea |
| 64. | Processed sweets procured from market | Sweets (Balushai/Ladoo/Malpua)  Jalebi |

**Supplementary File 3b**

**60 food sub-categories for winter season**

| **S.No.** | **Categories** | **Food Item Code** |
| --- | --- | --- |
| 1 | Commonly consumed non-indigenous cereal staples | Rice, Hybrid  Guhun/Wheat flour  Pioneer dhan  Diku baba |
| 2 | Less-commonly consumed non-Indigenous cereals | Corn/Makkai, Hybrid |
| 3 | Non-Indigenous Millets | Bajra/Gangayi |
| 4 | Non-Indigenous Cereals eaten as a snack | Muri/Puffed rice  Rice flakes/chirwa |
| 5 | Commonly consumed indigenous cereal staples | Laaldhan/Arababa  Safeddhan  Jedengdhan  Karangadhan  Gilti baba  Pundi goda  Rieci baba  Karnidhan  Lalatdhan  Panchanwayedhan  Dusridhan  Sambalpuri  Garmi dhan/teba baba  Pagla Goda  Sorno baba  Gudlu  Mahua dhan  Mota dhan  Munadhan  Maize |
| 6 | Less-commonly consumed Indigenous cereals | Daanidhan  Pasoda baba  Kannaudhan  Raasdhan  Minjri  Aara/Aarodhan  Jolpo baba  Hathipanjardhan  Rajdhan  Anthanawaye dhan  Naniha  Maniya dhan |
| 7 | Indigenous Millets | Bajra/Gangayi  Mandua/Kodhe/Janhe |
| 8 | Commonly consumed non-indigenous pulses | Raher/Red gram (whole)  Raher/Red gram (dehusked)  Masoor dal  Moong/green gram (whole) |
| 9 | Less commonly consumed non-indigenous pulses | Boot/Bengal gram dal  Moong/green gram (dehusked)  Rambada dal/Black gram/Urad  Rambada dal/Black gram/Urad (dehusked) |
| 10 | Commonly consumed indigenous pulses | Kulthi  Rambada dal/Urad/Black gram (dehusked) |
| 11 | Less commonly consumed indigenous pulses | Suthro  Khesari dal  Baturi/Tiri riti  Danbudi/Ghangra  Raher/ Red gram (whole)  Raher/Red gram (Dehusked)  Boot/Bengal gram dal  Rambada dal/Urad/Black gram (Whole) |
| 12 | Milk and milk products from the market | Cow’s milk  Buffalo’s milk  Powder milk  Packet milk |
| 13 | Non-indigenous meat products from reared livestock | Chicken  Eggs |
| 14 | Non-indigenous meat products from market | Meat/Mutton  Beef  Pig  Khasi |
| 15 | Indigenous meat products from forest/field/open spaces | Rabbit  Squirrel  Jangli murgi  Jungli Peacock  Tumbuli (insect)  Neelirasi  Jangli suar  Porcupine/Saahi/Jikki/Torod  Bando  Hadhoga/Siyar  Pigeon/Kabutar  Bees/Madhumakkhi  Chitri  Duhur  Sursuri  Ghaghar  Maina  Putam  Demta  Askal  Burdliyad/Burdulu/Ufia  Dhamna  Haau (Laalchiti)  Gudu |
| 16 | Non-indigenous fish | Katla  Rohu |
| 17 | Commonly consumed indigenous fish from water bodies | Iccha/Chingri  Pothi  Koronjo  Bale  Budu  Sundi/Getumachli  Hadad hai/Chirpi machli  Chudhako/Chodha  Dry fish |
| 18 | Less commonly consumed indigenous fish from water bodies | Linda  Aira  Kakandahayi  Binghayi  Madsakam  Genger |
| 19 | Commonly consumed indigenous molluscs from water bodies | Setua/Keyosuti |
| 20 | Non-indigenous fruits from open spaces | Guava/Tamras  Amrit/Papaya  Custard Apple  Kundrum/Jojo Fruit |
| 21 | Non-indigenous fruits from market | Apple  Banana  Amla  Coconut  Grapes  Orange  Pomegranate |
| 22 | Indigenous fruits from forests and/or open spaces | Amda/Amru  Ber  Miril/Mirle  Koa  Banyan fruit |
| 23 | Commonly consumed non-indigenous GLV cultivated | Mustard leaves/Maani arak  Onion leaves  Bathua leaves |
| 24 | Commonly consumed non-indigenous GLV from market | Muri arak/Radish greens  China palak  Palak |
| 25 | Non-indigenous GLV consumed as condiments/herbs | Mint  Coriander |
| 26 | Commonly consumed Indigenous GLV obtained from forest | Saru arak/kaanda arak  Sarla/Serali arak  Mattha saag  Sanai saag |
| 27 | Less commonly consumed Indigenous GLV obtained from forest | Heteteyo arak  Biyur arak |
| 28 | Commonly consumed Indigenous GLV growing as weeds | Mattha saag  Chatom arak |
| 29 | Commonly consumed Indigenous GLV cultivated | Munga arak/sajna  Chiringid arak  Himti/Mui arak  Chana saag/Bengal gram leaves  Amaranth/Leper arak  Chakod/choke ara  Kudrum saag/jojo arak  Sweet potato leaves  Pumpkin leaves |
| 30 | Less commonly consumed Indigenous GLV cultivated | Aloo arak  Lehsun saag  Chaari arak  Khesari saag  Posta arak  Teeri-riti arak  Losodar arak  Muchdi ara |
| 31 | Non-indigenous vegetables from market | Cauliflower  Tomato  Bitter Gourd/Karela  Bengar/Brinjal  Cucumber  Lady’s finger  Cabbage  Bottle gourd |
| 32 | Non-indigenous vegetables cultivated | Papaya/Man kunda  Pumpkin/Kumda/Kaddu/Kohda |
| 33 | Less commonly consumed indigenous vegetables from forests | Bir karela  Hutarba  Burju baha |
| 34 | Commonly consumed Indigenous vegetables from market | Kundri/Kundru  Ridge gourd/Jhinga  Dodo  Field beansMalhan/Sem/Simbi |
| 35 | Commonly consumed Indigenous vegetables cultivated | Sanai/Jindiba phool  Kundrum flower |
| 36 | Less commonly consumed indigenous vegetables cultivated | Barbatti vegetable/Bodi |
| 37 | Non-indigenous roots and tubers as a vegetable preparation from market | Ridge gourd/Jhinga  Dodo |
| 38 | Non-indigenous roots and tubers used as a condiment from market | Garlic  Onion |
| 39 | Non-indigenous roots and tubers cultivated | Carrot |
| 40 | Commonly consumed indigenous tubers from forest | Jat sanga  Saru sanga |
| 41 | Less commonly consumed indigenous tubers from forest | Adel sanga  Haseaar sanga  Maisarsanga  Koolarumpa |
| 42 | Commonly consumed indigenous tubers cultivated | Kondi sanga  Ole |
| 43 | Less commonly consumed indigenous tubers cultivated | Marom sanga |
| 44 | Non-indigenous saturated oils and fats from market | Ghee (Cow/Buffalo)  Vanaspati/Dalda |
| 45 | Non-indigenous unsaturated oils from market | Refined vegetable oil |
| 46 | Non-indigenous unsaturated Oils derived from pressing seeds | Groundnut oil  Mustard oil |
| 47 | Home produced indigenous oils and fats | Turi/Desi mustard oil  Coconut oil  Kusum oil  Surgunja oil |
| 48 | Nuts and oilseeds from Govt. programme | Groundnuts |
| 49 | Nuts and oilseeds from market | Coconuts |
| 50 | Sugars from forest | Honey |
| 51 | Sugars from market | Brown sugar  Jaggery |
| 52 | Sugars from TPDS | Sugar |
| 53 | Alcohols prepared locally | Handiya  Khijur Taadi  Mahua Taadi  Tukui Handi |
| 54 | Alcohols bought from the market | English |
| 55 | Miscellaneous foods eaten as a side dish | Pickle |
| 56 | Cooked Sweets and desserts | Kheer |
| 57 | Freshly prepared dishes from the market | Muri ghugni  Aloo Chop/Samosa/Dhuska/Idli  Aloo Pakora/Bread Pakora  Chowmein |
| 58 | Packaged foods | Pav roti  Bhujia/Mixture/Papad/Sev  Chips/Kachori/Mathri/Namkeen/Nimki  Biscuit, sweet  Biscuit, salty  Chocolate |
| 59 | Hot beverage | Tea |
| 60 | Processed sweets procured from market | Sweets (Balushai/Ladoo/Malpua/Gulgula)  Jalebi |
