# Supplementary material for: Indigenous food environment and dietary patterns of Munda community of Jharkhand, India
Source: BMC Nutr. 2025 Oct 21;11:189. doi: 10.1186/s40795-025-01159-2 (PMC12539013; doi:10.1186/s40795-025-01159-2)
Supplement: Supplementary file 4 — Supplementary Material 4 [file 40795_2025_1159_MOESM4_ESM.docx]

**Supplementary file 4. Association of various dietary patterns followed in the monsoon season with socio-demographic factors (logistic regression)**

| **Characteristic** | **Odds ratio (95% CI)^1^, p-value** | | |
| --- | --- | --- | --- |
|  | **Market-dominant dietary pattern** | **Nature-dominant**  **dietary pattern** | **Mixed-source dietary pattern** |
| Wealth score (n=141) | 1.07 (0.89, 1.28)  0.46 | 0.98 (0.81, 1.17)  0.80 | 0.91 (0.77, 1.09)  0.31 |
| Type of Family (n=141)  Joint/Extended  Nuclear | Ref   - 1. (0.54,1.91)   0.97 | Ref  1.52 (0.81, 2.88)  0.19 | Ref  1.18 (0.63, 2.22)  0.61 |
| Number of family members (n=142) | 0.90 (0.78,1.03)  0.14 | 1.04 (0.90, 1.20)  0.62 | 1.09 (0.95, 1.27)  0.22 |
| Education of the HoH (n=141)  No formal education  Primary schooling and below  Secondary schooling and above | Ref  1.03 (0.51, 2.08)  1.66 (0.74, 3.75)  0.45 | Ref  0.65 (0.32, 1.32)  0.45 (0.20, 0.98)  0.12 | Ref  1.05 (0.52, 2.14)  1.36 (0.63, 2.98)  0.73 |
| Occupation of HoH (n=141)  Other  Settled agriculture | Ref  2.09 (1.01, 4.40)  **0.048** | Ref  0.74 (0.35, 1.54)  0.43 | Ref  1.19 (0.56, 2.55)  0.65 |
| Gender of HoH (n=142)  Male  Female | Ref  1.12 (0.57, 2.19)  0.74 | Ref  0.94 (0.46, 1.92)  0.87 | Ref  1.83 (0.89, 3.82)  0.10 |
| Distance to nearest market (n=147)  <1 km  1-3 km  3-5 km  >5 km | Ref  1.17 (0.46, 2.97)  1.17 (0.45, 3.06)  0.70 (0.24, 2.03)  0.66 | Ref  0.65 (0.26, 1.59)  0.58 (0.23, 1.47)  0.67 (0.23, 1.97)  0.72 | Ref  1.16 (0.49, 2.79)  1.43 (0.56, 3.65)  1.95 (0.68, 5.67)  0.59 |
| Collect food items from forest (n=147)  No  Yes | Ref  0.86 (0.45, 1.64)  0.64 | Ref  1.08 (0.56, 2.07)  0.83 | Ref  0.94 (0.48, 1.87)  0.87 |
| Distance to nearest forest (n=107)  <1 km  1-3 km  >3 km | Ref  1.18 (0.53, 2.67)  1.84 (0.67, 5.15)  0.48 | Ref  1.24 (0.54, 2.85)  1.76 (0.66, 4.75)  0.53 | Ref  0.92 (0.41, 2.05)  0.87 (0.33, 2.30)  0.96 |
| Access food items from pond/river/small streams (n=147)  No  Yes | Ref  1.00 (0.53, 1.89)  >0.99 | Ref  1.36 (0.7, 2.68)  0.37 | Ref  1.52 (0.77, 3.0)  0.23 |
| Possess/share domestic animals (n=147)  No  Yes | Ref  1.26 (0.46, 3.50)  0.66 | Ref  0.84 (0.3, 2.3)  0.74 | Ref  1.09 (0.39, 3.07)  0.87 |
| Food Access Diversity Index (n=141) | 2.07 (0.64, 6.89)  0.23 | 0.85 (0.26, 2.79)  0.79 | 3.93 (1.13, 14.3)  **0.034** |

^1^Logistic regression was carried out to explore the association of different factors with household dietary patterns
