# Supplementary material for: Indigenous food environment and dietary patterns of Munda community of Jharkhand, India
Source: BMC Nutr. 2025 Oct 21;11:189. doi: 10.1186/s40795-025-01159-2 (PMC12539013; doi:10.1186/s40795-025-01159-2)
Supplement: Supplementary file 5 — Supplementary Material 5 [file 40795_2025_1159_MOESM5_ESM.docx]

**Supplementary file 5.** **Association of various dietary patterns followed in the winter season with socio-demographic factors (logistic regression)**

| **Characteristic** | **Odds ratio (95% CI)^1^, p-value** | | |
| --- | --- | --- | --- |
|  | **Nature-procured dietary pattern** | **Mixed-source dietary pattern** | **Market-dominant dietary pattern** |
| Wealth score (n=94) | 1.00 (0.77, 1.31)  0.97 | 0.80 (0.61, 1.03)  0.087 | 0.78 (0.60, 1.01)  0.062 |
| Type of Family (n=94)  Joint/Extended  Nuclear | Ref  2.62 (1.21, 5.82)  **0.016** | Ref  0.54 (0.25, 1.15)  0.11 | Ref  2.90 (1.34, 6.47)  **0.008** |
| Number of family members (n=91) | 1.07 (0.88,1.30)  0.51 | 1.02 (0.85, 1.24)  0.81 | 1.04 (0.86, 1.27)  0.69 |
| Education of the HoH (n=94)  No formal education  Primary schooling and below  Secondary schooling and above | Ref  2.27 (0.95, 5.57)  0.50 (0.17, 1.40)  0.033 | Ref  0.82 (0.35, 1.95)  0.44 (0.15, 1.23)  0.30 | Ref  0.77 (0.33, 1.79)  0.48 (0.17, 1.34)  0.38 |
| Occupation of HoH (n=94)  Other  Settled agriculture | Ref  0.47 (0.16, 1.31)  0.16 | Ref  1.27 (0.5, 3.25)  0.61 | Ref  0.71 (0.27, 1.84)  0.48 |
| Gender of HoH (n=91)  Male  Female | Ref  0.43 (0.16, 1.14)  0.093 | Ref  1.46 (0.56, 3.90)  0.44 | Ref  0.52 (0.19, 1.34)  0.18 |
| Distance to nearest market (n=97)  <1 km  1-3 km  3-5 km  >5 km | Ref  2.62 (0.79, 9.34)  4.70 (1.42, 16.9)  2.18 (0.58, 8.65)  0.009 | Ref  2.24 (0.68, 7.70)  1.80 (0.55, 6.11)  1.69 (0.43, 6.84)  0.63 | Ref  1.69 (0.52, 5.79)  3.64 (1.12, 12.5)  1.49 (0.39, 5.84)  0.12 |
| Collect food items from forest (n=94)  No  Yes | Ref  0.74 (0.33, 1.67)  0.47 | Ref  1.66 (0.75, 3.73)  0.21 | Ref  0.72 (0.31, 1.66)  0.45 |
| Distance to nearest forest (n=67)  <1 km  1-3 km  >3 km | Ref  1.12 (0.39, 3.23)  1.28 (0.36, 4.52)  0.93 | Ref  1.18 (0.40, 3.44)  0.34 (0.09, 1.21)  0.10 | Ref  1.79 (0.59, 5.65)  1.38 (0.41, 4.77)  0.59 |
| Access food items from pond/river/small streams (n=93)  No  Yes | Ref  1.11 (0.46, 2.71)  0.82 | Ref  0.49 (0.19, 1.24)  0.14 | Ref  0.88 (0.38, 2.06)  0.77 |
| Possess/share domestic animals (n=93)  No  Yes | Ref  0.59 (0.17, 1.95)  0.39 | Ref  1.32 (0.39, 4.62)  0.65 | Ref  1.62 (0.50, 5.54)  0.42 |
| Food Access Diversity Index (n=94) | 0.61 (0.16, 2.33)  0.47 | 0.36 (0.10, 1.30)  0.12 | 0.59 (0.14, 2.33)  0.45 |

^1^Logistic regression was carried out to explore the association of different factors with household dietary patterns
